# Glycocalyx dysregulation impairs blood–brain barrier in ageing and disease

**DOI:** 10.1038/s41586-025-08589-9

**Published:** 2025-02-26

**Authors:** Sophia M. Shi, Ryan J. Suh, D. Judy Shon, Francisco J. Garcia, Josephine K. Buff, Micaiah Atkins, Lulin Li, Nannan Lu, Bryan Sun, Jian Luo, Ning-Sum To, Tom H. Cheung, M. Windy McNerney, Myriam Heiman, Carolyn R. Bertozzi, Tony Wyss-Coray

**Affiliations:** 1 Department of Chemistry, Stanford University, Stanford, CA USA; 2 Stanford Chemistry, Engineering and Medicine for Human Health (ChEM-H), Stanford University, Stanford, CA USA; 3 Department of Neurology and Neurological Sciences, Stanford University School of Medicine, Stanford, CA USA; 4 Wu Tsai Neurosciences Institute, Stanford University School of Medicine, Stanford, CA USA; 5 Picower Institute for Learning and Memory, Cambridge, MA USA; 6 Department of Brain and Cognitive Sciences, MIT, Cambridge, MA USA; 7 The Phil and Penny Knight Initiative for Brain Resilience, Stanford University, Stanford, CA USA; 8 Palo Alto Veterans Institute for Research, Palo Alto, CA USA; 9 Hong Kong Center for Neurodegenerative Diseases, Hong Kong, China; 10 Division of Life Science, Center for Stem Cell Research, HKUST-Nan Fung Life Sciences Joint Laboratory, State Key Laboratory of Molecular Neuroscience, Daniel and Mayce Yu Molecular Neuroscience Center, The Hong Kong University of Science and Technology, Hong Kong, China; 11 Department of Psychiatry, Stanford University School of Medicine, Stanford, CA USA; 12 MIRECC, Department of Veterans Affairs, Palo Alto, CA USA; 13 Howard Hughes Medical Institute, Stanford University, Stanford, CA USA

**Keywords:** Blood-brain barrier, Glycobiology, Neurodegenerative diseases

## Abstract

The blood–brain barrier (BBB) is highly specialized to protect the brain from harmful circulating factors in the blood and maintain brain homeostasis^1,2^. The brain endothelial glycocalyx layer, a carbohydrate-rich meshwork composed primarily of proteoglycans, glycoproteins and glycolipids that coats the BBB lumen, is a key structural component of the BBB^3,4^. This layer forms the first interface between the blood and brain vasculature, yet little is known about its composition and roles in supporting BBB function in homeostatic and diseased states. Here we find that the brain endothelial glycocalyx is highly dysregulated during ageing and neurodegenerative disease. We identify significant perturbation in an underexplored class of densely O-glycosylated proteins known as mucin-domain glycoproteins. We demonstrate that ageing- and disease-associated aberrations in brain endothelial mucin-domain glycoproteins lead to dysregulated BBB function and, in severe cases, brain haemorrhaging in mice. Finally, we demonstrate that we can improve BBB function and reduce neuroinflammation and cognitive deficits in aged mice by restoring core 1 mucin-type O-glycans to the brain endothelium using adeno-associated viruses. Cumulatively, our findings provide a detailed compositional and structural mapping of the ageing brain endothelial glycocalyx layer and reveal important consequences of ageing- and disease-associated glycocalyx dysregulation on BBB integrity and brain health.

## Abstract (web summary)

Disruption of mucin-domain glycoprotein expression and function in the endothelial glycocalyx are associated with ageing and Alzheimer’s disease, leading to dysregulated blood–brain barrier function.

## Main

**Fig. 1:**
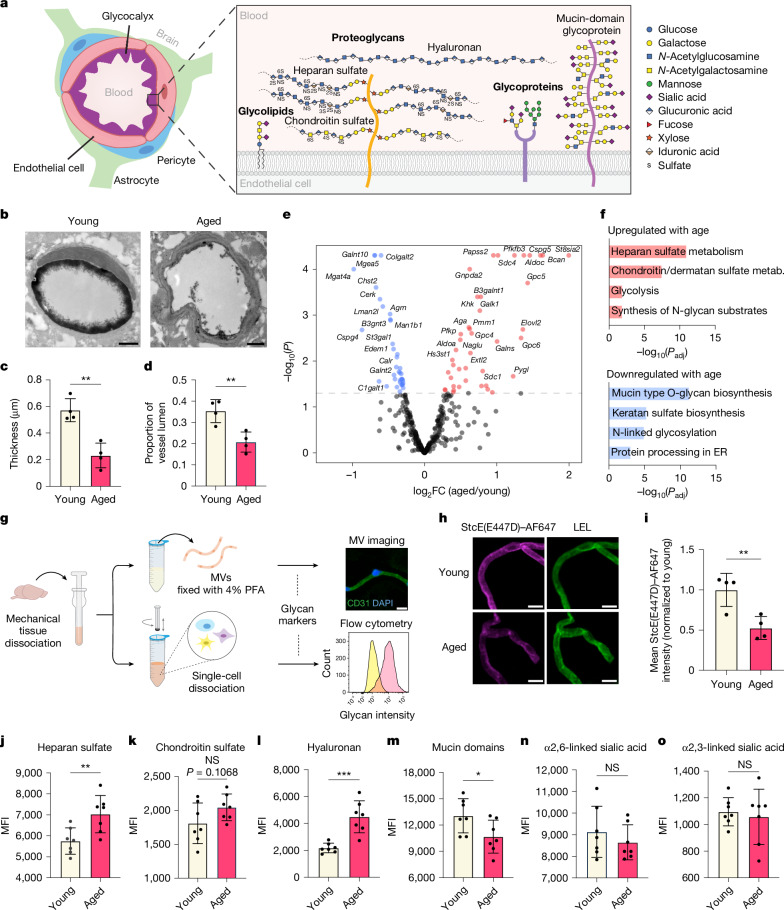
The brain endothelial glycocalyx is highly dysregulated during ageing. **a**, Diagram of the BBB and brain endothelial glycocalyx layer. Approximations used for the relative sizing of glycocalyx components are described in the Supplementary Notes. **b**, TEM of cortical capillaries with lanthanum nitrate staining from young (3-month-old) and aged (21-month-old) mice. Scale bars, 1 µm. **c**, Quantification of luminal endothelial glycocalyx thickness of young (3-month-old) and aged (21-month-old) mice (*n* = 4 mice per group; two-sided *t*-test; mean ± s.e.m.). **d**, Quantification of luminal endothelial glycocalyx area of young (3-month-old) and aged (21-month-old) mice (*n* = 4 mice per group; two-sided *t*-test; mean ± s.e.m.). **e**, Volcano plot of differentially expressed glycosylation-related genes in brain endothelial cells from young (3-month-old) and aged (19-month-old) mice (genes upregulated with age in red and genes downregulated with age in blue). Original bulk RNA-seq data are from Yousef, et al.^12^. **f**, Top glycosylation-related pathways that are upregulated and downregulated with age in brain endothelial cells. ER, endoplasmic reticulum; metab., metabolism. **g**, Experimental scheme for glycan and glycoconjugate profiling of brain endothelial cells via microvessel (MV) imaging and flow cytometry. PFA, paraformaldehyde. **h**, Mucin-domain glycoprotein expression and *Lycopersicon esculentum* (tomato) lectin (LEL; endothelial marker) labelling in acutely isolated microvessels. Scale bars, 10 µm. AF647, Alexa Fluor 647. **i**, Quantification of **h** (*n* = 4 mice per group; two-sided *t*-test; mean ± s.e.m.). **j**–**o**, Median fluorescence intensity (MFI) of heparan sulfate (**j**; via 10E4 antibody), chondroitin sulfate (**k**; via CS-56 antibody), hyaluronan (**l**; via HABP), mucin-domain glycoproteins (**m**; via StcE(E447D)–AF647), α2,6-linked sialic acids (**n**; via SNA) and α2,3-linked sialic acids (**o**; via MAAII) on mechanically isolated brain endothelial cells from young (3-month-old) and aged (21-month-old) mice (*n* = 7 mice per group; two-sided *t*-test; mean ± s.e.m.). NS, not significant.

The BBB forms a tightly regulated vascular interface between the blood and brain that is essential for supporting proper brain function. The highly restrictive nature of the BBB is enabled by the unique properties of brain endothelial cells, including specialized tight junctions, exceptionally low rates of fluid-phase transcytosis, and selective influx and efflux transporters^1,2^. The contribution of the brain endothelial glycocalyx layer to BBB function is increasingly being recognized^3,4^. The glycocalyx layer is a complex meshwork of glycans and glycoconjugate species (proteoglycans, glycoproteins and glycolipids) that coats the luminal surface of the BBB and, more broadly, mediates many cell surface processes including cell signalling, adhesion, transport and morphology^3,5,6^ (Fig. 1a). Despite forming the first barrier to entry through the BBB for any blood-derived factors, little is known about the composition and functional roles of the brain endothelial glycocalyx layer. Furthermore, BBB dysfunction, often characterized by increased vascular leakiness to neurotoxic and inflammatory circulating factors, is a key pathological hallmark in ageing and neurodegenerative diseases^2,7–9^. However, there is a lack of studies investigating the role of the glycocalyx in this process. Here we structurally and compositionally profile ageing- and disease-associated changes in the brain endothelial glycocalyx to determine how glycocalyx dysregulation in these states may contribute to BBB dysfunction.

## Glycocalyx dysregulation during ageing

**Extended Data Fig. 1:**
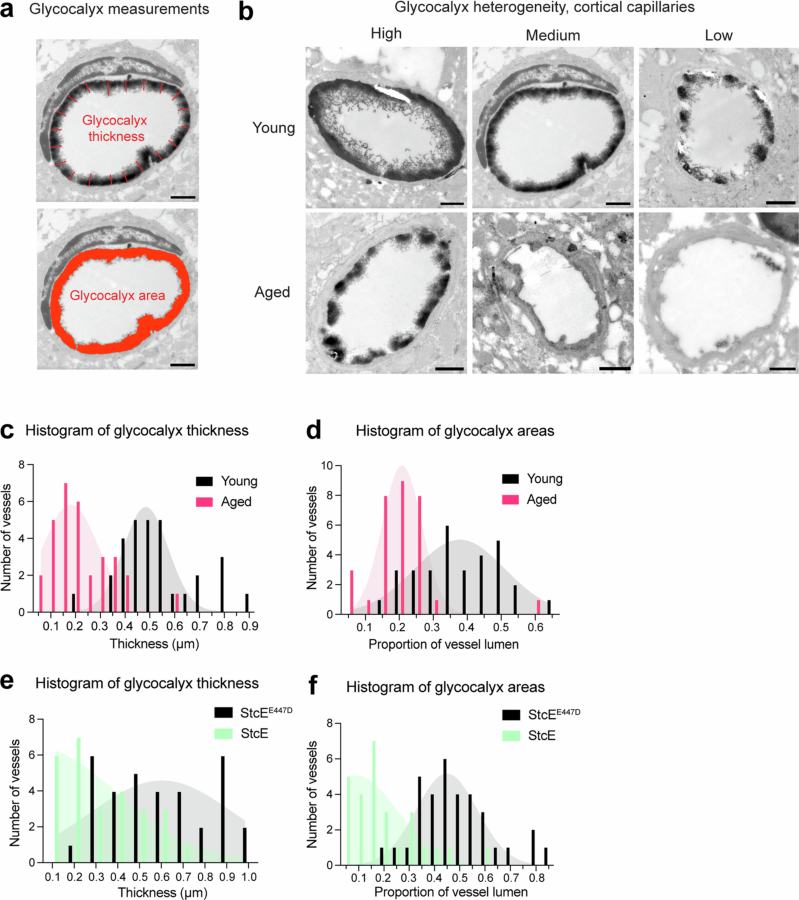
Heterogeneity in the brain endothelial glycocalyx layer. a) Diagram of glycocalyx thickness and area measurements. b) Transmission electron micrographs of cortical capillaries with lanthanum nitrate staining from young (3-month-old) and aged (21-month-old) mice. Representative images of capillaries with glycocalyx measurements approximately in the high (80^th^–90^th^ percentiles), medium (45^th^–55^th^ percentiles), and low (10^th^–20^th^ percentiles) ranges of all vessels captured for each group. Scale bar = 1 µm. c) Histograms of luminal endothelial glycocalyx thickness including 5-6 vessels from each animal in Fig. 1c. Fitted gaussian distributions are displayed for each group. d) Histograms of luminal endothelial glycocalyx areas including 5-6 vessels from each animal in Fig. 1d. Fitted gaussian distributions are displayed for each group. e) Histograms of luminal endothelial glycocalyx thickness including 5-6 vessels from each animal in Fig. 2d. Fitted gaussian distributions are displayed for each group. f) Histograms of luminal endothelial glycocalyx areas including 5-6 vessels from each animal in Fig. 2e. Fitted gaussian distributions are displayed for each group.

For initial characterization of ageing-associated bulk structural changes in the brain endothelial glycocalyx layer, we used transmission electron microscopy (TEM) with lanthanum nitrate staining to visualize brain endothelial glycocalyx layers in young (3-month-old) and aged (21-month-old) mice. Whereas traditional electron microscopy of the BBB does not enable visualization of the glycocalyx layer, incorporation of cationic metal stains, such as lanthanum nitrate or ruthenium red, into samples reveals a substantial layer of glycans on the vascular lumen^4,10,11^ (Fig. 1b). We observed a significant reduction in luminal glycocalyx layers in cortical capillaries of aged mice compared with those of young mice (Fig. 1b–d and Extended Data Fig. 1a–d). Quantitative analyses showed decreases in both average glycocalyx thickness (0.540 ± 0.086 μm versus 0.232 ± 0.092 μm) and average glycocalyx area (0.367 ± 0.054 versus 0.207 ± 0.047 as a proportion of lumen area) with ageing (Fig. 1c,d).

To identify classes of glycoconjugates (Fig. 1a) that might contribute to these ageing-associated aberrations in the glycocalyx, we analysed a previous bulk RNA-sequencing (RNA-seq) dataset from our laboratory of brain endothelial cells collected from young (3-month-old) and aged (19-month-old) mice^12^. We identified significant dysregulation of many glycosylation-related genes with ageing (Fig. 1e). Pathway analysis revealed notable upregulation of genes involved in heparan sulfate metabolism (*Sdc4*, *Hs3st1*, *Extl2* and *Gpc5*) and downregulation of genes involved in mucin-type O-glycan biosynthesis (*Galnt10*, *B3gnt3*, *Galnt2* and *C1galt1*) in aged brain endothelial cells compared with young brain endothelial cells (Fig. 1e,f).

**Extended Data Fig. 2:**
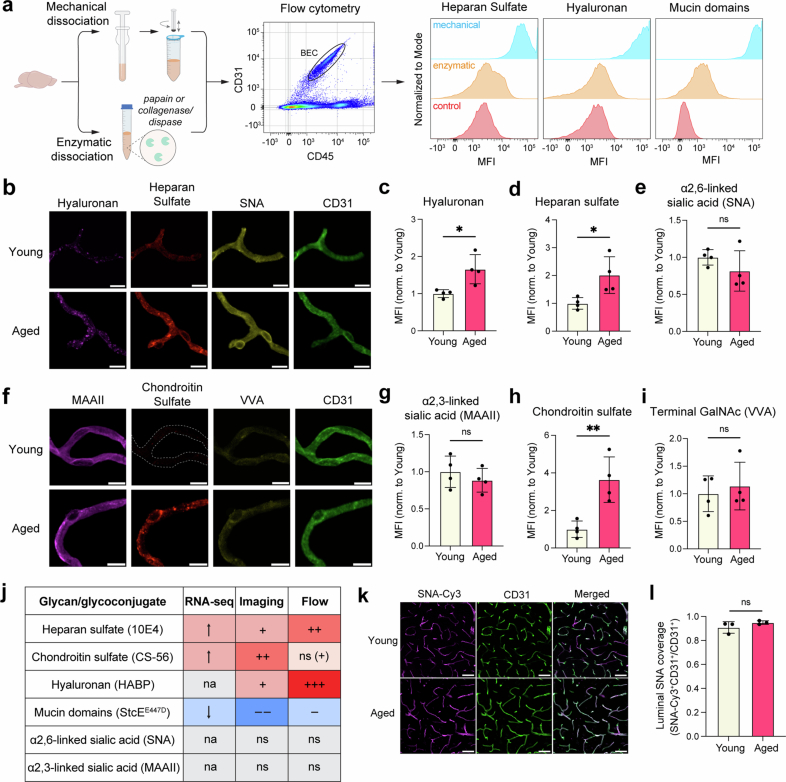
Compositional changes in the brain endothelial glycocalyx during aging. a) Flow cytometry experimental scheme comparing mechanical (blue) and enzymatic (orange) brain dissociation methods demonstrating that mechanical dissociation better preserves several classes of glycans/glycoconjugates on brain endothelial cells. b) Hyaluronan, heparan sulfate, and α2,6-linked sialic acid expression in acutely isolated microvessels from young (3-month-old) and aged (21-month-old) mice. Scale bar = 10 µm. c-e) Quantification of (b) (n = 4, two-sided t-test; mean ± s.e.m.). f) α2,3-linked sialic acid, chondroitin sulfate, and terminal GalNAc expression in acutely isolated microvessels from young (3-month-old) and aged (21-month-old) mice. Scale bar = 10 µm. g-i) Quantification of (f) (n = 4, two-sided t-test; mean ± s.e.m.). j) Summary of aging-associated changes in brain endothelial glycan/glycoconjugate classes observed using bulk RNA-seq, fluorescence microvessel imaging, and flow cytometry profiling. k) Luminal sialic acid expression based on SNA-Cy3 labeling in CD31^+^ cortical vasculature. Scale bar = 20 µm. l) Quantification of (k) (n = 3 mice per group; two-sided t-test; mean ± s.e.m.).

To determine whether these transcriptional changes were reflected at the level of cell surface glycosylation, we directly profiled brain endothelial cell glycosylation via imaging and flow cytometry-based assays using a panel of antibodies and binding proteins that are selective for several major classes of glycans, glycoconjugates and specific monosaccharides (Fig. 1g). The extensive extracellular matrix of the brain precluded reliable endothelial-specific quantification of many glycan and glycoconjugate species in intact brain slices using traditional immersion staining methods. Consequently, we optimized methods to dissociate brain tissue into microvessels or single cells and consistently found that mechanical dissociation methods better preserved brain endothelial glycocalyx staining compared with commonly used enzymatic dissociation methods^12–14^ (Fig. 1g and Extended Data Fig. 2a). Fluorescence imaging of acutely isolated microvessels from young and aged mice revealed significantly increased expression of hyaluronan (via hyaluronan binding protein (HABP)), heparan sulfate (via 10E4 antibody) and chondroitin sulfate (via CS-56 antibody), significantly decreased expression of mucin-domain glycoproteins (via StcE(E447D)), and no significant change in α2,6- or α2,3-linked sialic acids (via *Sambucus nigra* agglutinin (SNA) and *Maackia amurensis* agglutinin II (MAAII), respectively) or terminal *N*-acetylgalactosamine (via *Vicia villosa* agglutinin (VVA)) with ageing (Fig. 1h,i and Extended Data Fig. 2b–i). Flow cytometry analysis of acutely isolated brain endothelial cells demonstrated congruent results, which were also largely consistent with the dysregulated glycosylation pathways identified in our RNA-seq analysis (Fig. 1j–o and Extended Data Fig. 2j).

Together, these data demonstrate that brain endothelial cell surface glycosylation undergoes substantial structural and compositional alteration during ageing, which may in part be dictated by transcriptional changes. Of note, hyaluronan and heparan sulfate have previously been reported to be increased on the vasculature in ageing and neurodegenerative disease states and are commonly associated with negative consequences on BBB function and Alzheimer’s disease pathology^15–19^. However, to our knowledge, downregulation of mucin-type O-glycosylation has not previously been linked to ageing-associated BBB impairment, so we pursued this finding further.

## Downregulated mucin-type O-glycosylation

**Fig. 2:**
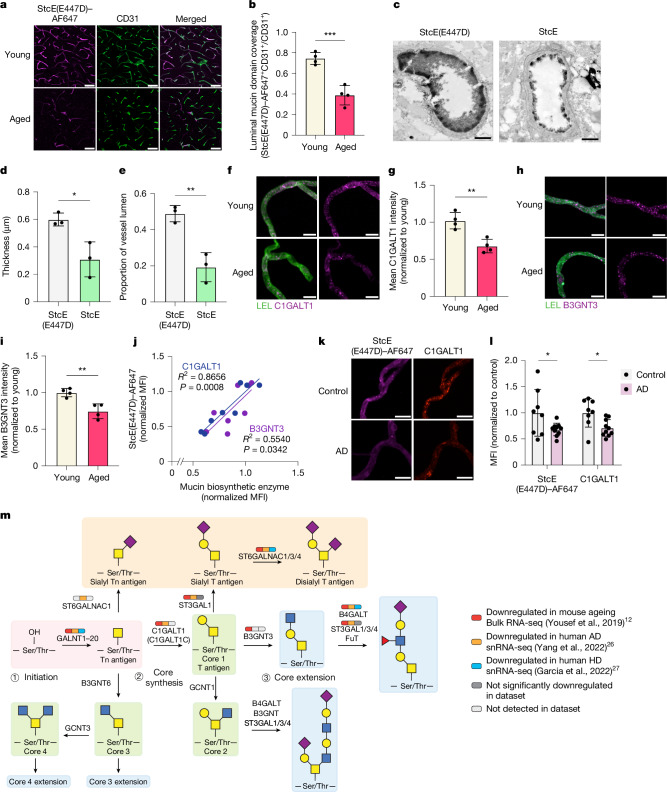
Mucin-type O-glycosylation is downregulated in brain endothelial cells during ageing and neurodegenerative disease. **a**, Luminal mucin-domain glycoprotein expression based on StcE(E447D)–AF647 labelling in CD31^+^ cortical vasculature. Scale bars, 20 µm. **b**, Quantification of **a** (*n* = 4 mice per group; two-sided *t*-test; mean ± s.e.m.). **c**, TEM of cortical capillaries with lanthanum nitrate staining from mice treated with StcE(E447D) or StcE for 24 h. Scale bars, 1 µm. **d**, Quantification of luminal endothelial glycocalyx thickness of mice treated with StcE(E447D) or StcE (*n* = 3 mice per group; two-sided *t*-test; mean ± s.e.m.). **e**, Quantification of luminal endothelial glycocalyx areas of mice treated with StcE(E447D) or StcE (*n* = 3 mice per group; two-sided *t*-test; mean ± s.e.m.). **f**, C1GALT1 expression in acutely isolated microvessels labelled with LEL. Scale bars, 10 µm. **g**, Quantification of **f** (*n* = 4 mice per group, two-sided *t*-test; mean ± s.e.m.). **h**, B3GNT3 expression in acutely isolated microvessels labelled with LEL. Scale bars, 10 µm. **i**, Quantification of **h** (*n* = 4 mice per group, two-sided *t*-test; mean ± s.e.m.). **j**, Linear correlation between StcE(E447D) and C1GALT1 (blue) or B3GNT3 (purple) expression in acutely isolated microvessels. **k**, C1GALT1 and mucin-domain glycoprotein expression in acutely isolated microvessels from Alzheimer’s disease (AD) and age-matched control brains. Scale bars, 10 µm. **l**, Quantification of **m** (*n* = 8 control and 10 Alzheimer’s disease samples, two-sided *t*-test; mean ± s.e.m.). **m**, Mucin-type O-glycan biosynthetic pathway. Broad transcriptional downregulation of brain endothelial core 1 mucin-type O-glycan biosynthetic enzymes is observed in mouse ageing, Alzheimer’s disease and Huntington’s disease (HD) RNA-seq datasets^12,26,27^.

**Extended Data Fig. 3:**
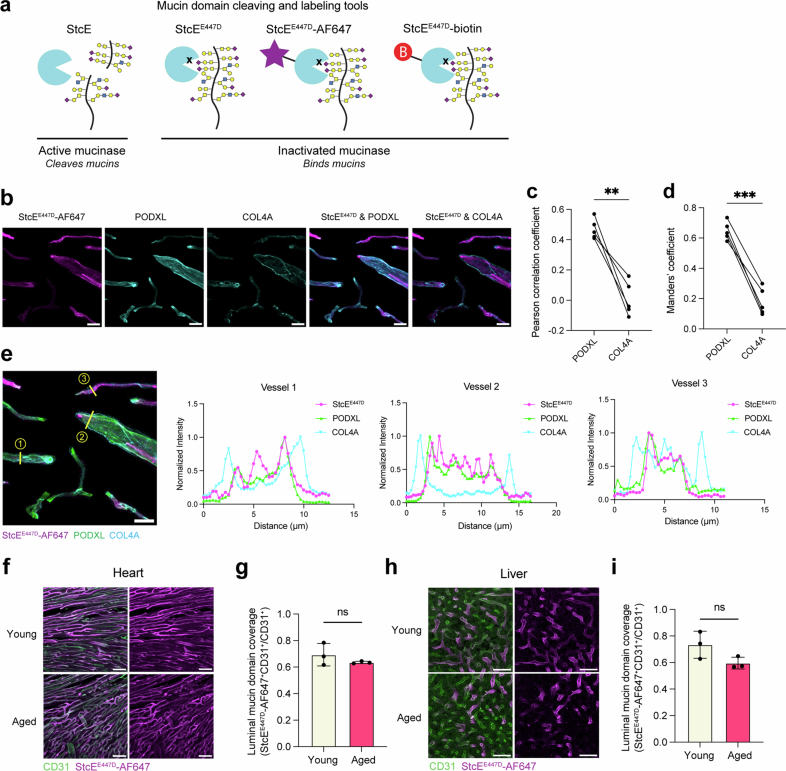
Mucinase-derived tools for mucin degradation and vascular labeling. a) Diagram of mucin degrading and labeling tools used in experiments. b) StcE^E447D^-AF647, PODXL (luminal marker), and COL4A (basement membrane marker) labeling in cortical vessels. Scale bar = 20 µm. c) Quantification of StcE^E447D^-AF647 colocalization with PODXL and COL4A via Pearson correlational analyses (n = 5 mice; two-sided paired t-test; mean ± s.e.m.). d) Quantification of StcE^E447D^-AF647 colocalization with PODXL and COL4A via Mander’s correlational analyses (n = 5 mice; two-sided paired t-test; mean ± s.e.m.). e) Marker intensity profiles across three vessels showing spatial correlation between StcE^E447D^-AF647, PODXL, and COL4A. Yellow labels indicate where intensity profiles were taken in representative image. Scale bar = 20 µm. f) Luminal mucin-domain glycoprotein expression in CD31^+^ vasculature in the hearts of young (3-month-old) and aged (21-month-old) mice. Scale bar = 20 µm. g) Quantification of (f) (n = 3 mice per group; two-sided t-test; mean ± s.e.m.). h) Luminal mucin-domain glycoprotein expression in CD31^+^ vasculature in the livers of young (3-month-old) and aged (21-month-old) mice. Scale bar = 20 µm. i) Quantification of (h) (n = 3 mice per group; two-sided t-test; mean ± s.e.m.).

Mucin-type O-glycosylation is a post-translational modification consisting of glycans initiated by an α-*N*-acetylgalactosamine (α-GalNAc) attached to proteins via serine and threonine residues. Regions of dense mucin-type O-glycosylation create mucin domains that adopt extended bottlebrush-like structures with distinct biophysical and biochemical properties. Although these domains are classically studied in the canonical mucin (MUC) family of proteins, which are highly expressed by mucosal epithelial cells, there are many mucin domain-containing glycoproteins beyond the MUC family that have widespread tissue expression and mediate diverse cell surface processes, including modulation of membrane morphology, signalling and cell–cell interactions^6,20–22^. Although this class of glycoproteins has not collectively been studied on the brain vasculature, many mucin-domain glycoproteins are abundantly expressed in brain endothelial cells including PODXL, CD34 and DAG1 (Supplementary Table. 1 and Supplementary Fig. 1). To evaluate the contribution of mucin-domain glycoproteins to the brain endothelial glycocalyx layer, we utilized a set of recombinant mucin-selective proteins derived from a broadly cleaving bacterial mucinase known as secreted protease of C1 esterase inhibitor (StcE)^22–24^ (Extended Data Fig. 3a). StcE has a distinct peptide- and glycan-based cleavage motif that is specific to mucin domains^23^. Leveraging this specificity, Alexa Fluor 647 (AF647)-conjugated catalytically inactivated StcE (StcE(E447D)–AF647) was previously developed as a mucin-selective staining reagent^22^ and was used in this study to label luminal mucin-domain glycoproteins, unconfounded by abluminal glycoproteins, via intracardial perfusion (Extended Data Fig. 3a–e). This technique revealed strong luminal mucin-domain glycoprotein staining in young mice and weaker, more heterogeneous staining in aged mice (Fig. 2a,b). Notably, this age-dependent decrease in luminal StcE(E447D)–AF647 signal appears to be particular to the brain; we observed little to no difference in luminal mucin-domain glycoprotein coverage in the heart or liver with ageing (Extended Data Fig. 3f–i). Despite some overlapping substrate specificity between StcE(E447D) and SNA, perfusion of Cy3-conjugated SNA (SNA–Cy3) showed no significant differences in luminal cerebrovascular labelling between young and aged mice (Extended Data Fig. 2k,l). Furthermore, intravenous injection of 0.25 mg kg^−1^ of enzymatically active StcE in young mice caused significant degradation of the brain endothelial glycocalyx layer after 24 h, as visualized using TEM with lanthanum nitrate staining (Fig. 2c–e and Extended Data Fig. 1e,f). These data demonstrate that mucin-domain glycoproteins are crucial structural constituents of the brain endothelial glycocalyx layer and suggest that their dysregulation may contribute to structural impairment of the glycocalyx layer observed with ageing (Fig. 1b).

**Extended Data Fig. 4:**
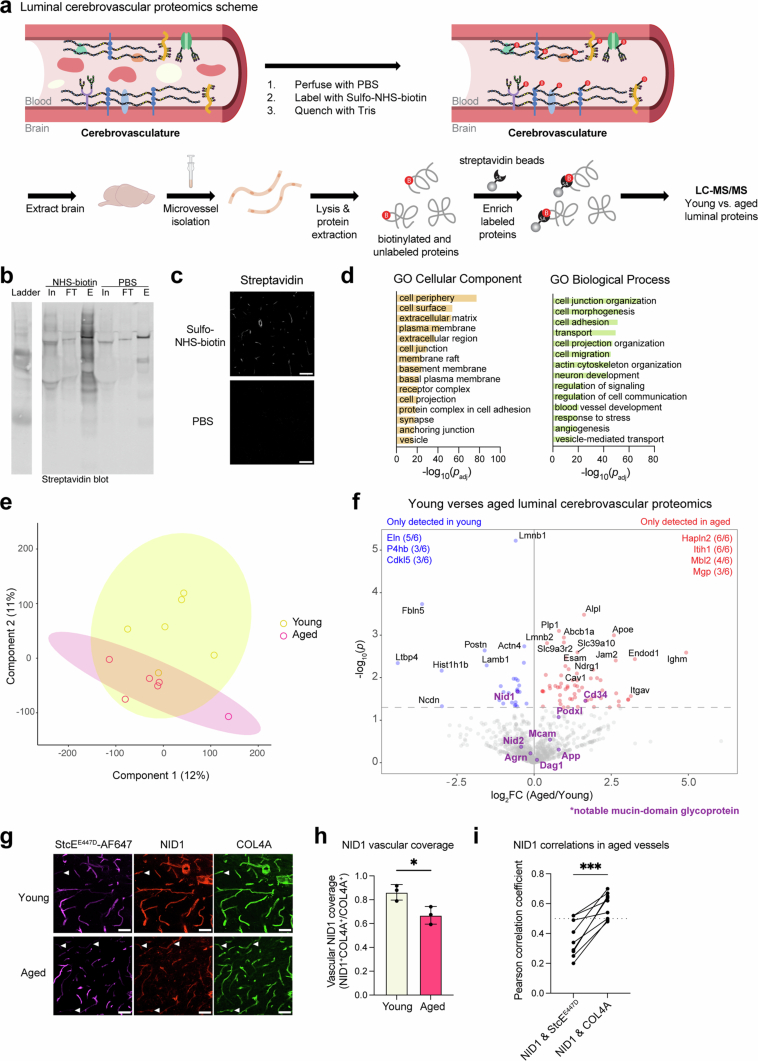
Luminal cerebrovascular proteomics does not reveal substantial downregulation of mucin-domain glycoprotein scaffolds with age. a) Luminal cerebrovascular proteomics scheme. b) Western blot of brain microvessel lysates from mice perfused with PBS or sulfo-NHS-biotin detected via streptavidin. In, input; FT, flowthrough; E, eluate. c) Cerebrovascular labeling by sulfo-NHS-biotin detected via streptavidin. Scale bar = 25 µm. d) Gene ontology (GO) term cellular component and biological process analysis of all proteins detected in luminal cerebrovascular proteomics experiment. e) Principal component analysis (PCA) of proteomic samples from young (3-month-old) versus aged (21-month-old) mice (n = 6 mice per group). f) Volcano plot of luminal cerebrovascular proteins identified in young (3-month-old) versus aged (21-month-old) mice (n = 6 mice per group). Peptide and protein identifications were filtered to a 1% FDR. Notable mucin-domain glycoproteins are highlighted in purple. Proteins only detected in young animals (in ≥ 50% of young animals) are highlighted in blue in the upper lefthand corner. Proteins only detected in aged animals (in ≥ 50% of aged animals) are highlighted in red in the upper righthand corner. g) NID1 expression in cortical vessels in young (3-month-old) versus aged (21-month-old) mice. Areas with poorly colocalized NID1 and StcE^E447D^-AF647 signal are indicated by the white triangles. Scale bar = 50 µm. h) Quantification of NID1 expression in COL4A^+^ vasculature in (g) (n = 3 mice per group; two-sided t-test; mean ± s.e.m.). i) Quantification of vascular NID1 colocalization with StcE^E447D^ and COL4A via Pearson correlation (n = 8 images from aged mouse group; two-sided paired t-test; mean ± s.e.m.).

The age-dependent loss of mucin-domain labelling via StcE(E447D)–AF647 could be owing to either a decrease in mucin-type O-glycosylation, as suggested by our bulk RNA-seq data (Fig. 1e,f), or a decrease in the mucin-domain glycoprotein scaffolds themselves. To test the latter, we compared the luminally enriched cerebrovascular proteome of young and aged mice by perfusing mice with membrane-impermeable sulfo-NHS-biotin to chemically modify luminal proteins with a biotin tag, which were subsequently enriched for mass spectrometry-based proteomic analysis (Extended Data Fig. 4a–c and Supplementary Fig. 2a–c). We identified 1,080 unique proteins, and Gene Ontology (GO) term analysis verified robust enrichment of proteins localized to the cell membrane and periphery and involved in cell surface processes including cell junction organization, adhesion and transport (Extended Data Fig. 4d–f and Supplementary Data 1). In addition, we observed upregulation of several brain endothelial cell membrane proteins that were previously known to be upregulated with ageing, including ALPL and CAV1^25^. However, NID1 was the only significantly downregulated known mucin-domain glycoprotein detected in our dataset, but the loss of luminal StcE(E447D)–AF647 signal in aged mice did not correlate with a loss of NID1 signal (Extended Data Fig. 4f–i). Although we are constrained by the availability of mucin-domain glycoprotein annotations and the limitations of liquid chromatography–tandem mass spectrometry (LC–MS/MS) detection, we did not find an obvious loss of mucin-domain glycoprotein scaffolds with age on the vascular lumen. By contrast, we observed significantly decreased levels of the mucin-type O-glycan biosynthetic enzymes C1GALT1 and B3GNT3 in acutely isolated cerebral microvessels from aged mice compared with those from young mice (Fig. 2f–i), consistent with our transcriptomic data. Levels of C1GALT1 and B3GNT3 also strongly correlated with StcE(E447D)–AF647 intensity in microvessels (Fig. 2j), further suggesting that downregulation of mucin-type O-glycan biosynthetic enzymes may contribute to the loss of mucin-domain glycoprotein staining observed with ageing.

**Extended Data Fig. 5:**
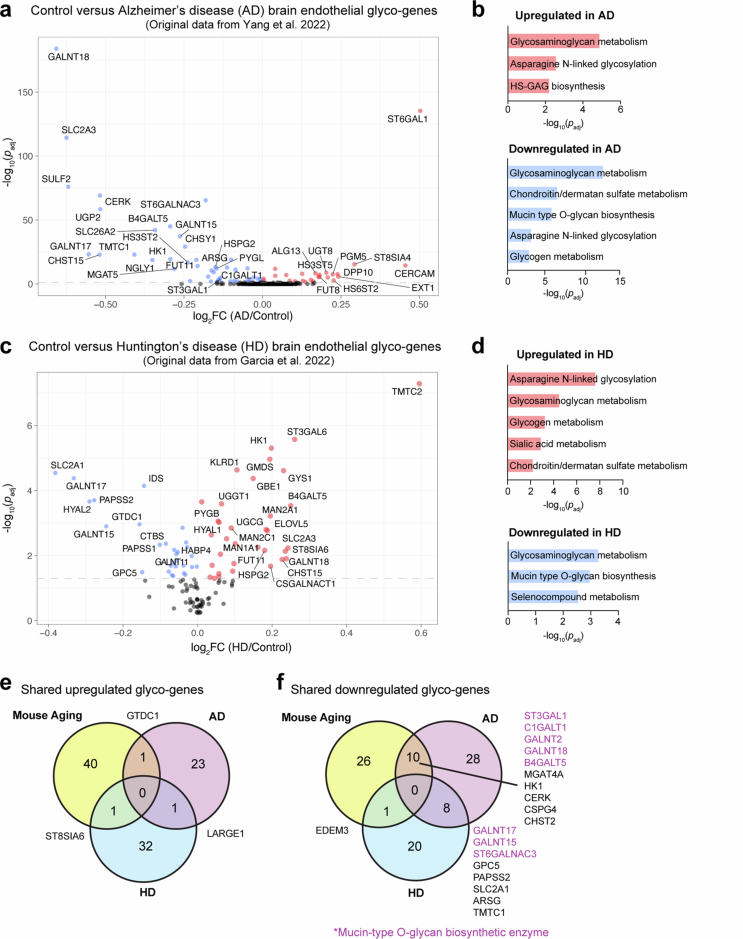
Transcriptional dysregulation in brain endothelial glycosylation pathways in neurodegenerative diseases. a) Volcano plot of differentially expressed glyco-genes in brain endothelial cells from patients with Alzheimer’s disease (AD) and age-matched control individuals (genes upregulated with AD in red and genes downregulated with AD in blue). Original snRNA-seq data from Yang, et al.^26^. b) Top upregulated and downregulated glycosylation-related pathways in brain endothelial cells in patients with AD versus age-matched control individuals. c) Volcano plot of differentially expressed glyco-genes in brain endothelial cells from patients with Huntington’s disease (HD) and age-matched control individuals (genes upregulated with HD in red and genes downregulated with HD in blue). Original snRNA-seq data from Garcia, et al.^27^. d) Top upregulated and downregulated glycosylation-related pathways in brain endothelial cells in patients with HD versus age-matched control individuals. e) Venn diagram of significantly upregulated brain endothelial glyco-genes that are shared amongst mouse aging, AD, and HD datasets. f) Venn diagram of significantly downregulated brain endothelial glyco-genes that are shared amongst mouse aging, AD, and HD datasets. Mucin-type O-glycan biosynthetic enzymes are highlighted in purple.

Considering that many of the same molecular changes associated with BBB dysfunction occur across different CNS disease states^7^, we sought to determine whether similar pathways of brain endothelial glycocalyx dysregulation seen in our ageing dataset could be observed in different neurodegenerative diseases. We conducted differential glycosylation-related gene analysis on previously published single-nucleus RNA-seq (snRNA-seq) datasets of Alzheimer’s disease^26^ and Huntington’s disease^27^ and found brain endothelial mucin-type O-glycan biosynthesis to be a shared downregulated pathway enriched in both disease datasets (Extended Data Fig. 5a–d). Whereas very few upregulated glycosylation-related genes overlapped between any two of the datasets (ageing, Alzheimer’s disease or Huntington’s disease), approximately one-third of all significantly downregulated glycosylation-related genes in any one dataset was shared by another dataset (Extended Data Fig. 5e,f). Out of the 19 shared downregulated genes, 8 of them were mucin-type O-glycan biosynthetic enzymes (Fig. 2m and Extended Data Fig. 5f). We subsequently confirmed decreased C1GALT1 and mucin-domain glycoprotein levels via staining in acutely isolated microvessels from brains of patients with Alzheimer’s disease compared with age-matched controls (Fig. 2k,l). These data suggest that downregulation of mucin-type O-glycosylation may contribute to a conserved glycosylation signature of BBB dysfunction seen across multiple neurodegenerative states.

## Vascular impairment

**Fig. 3:**
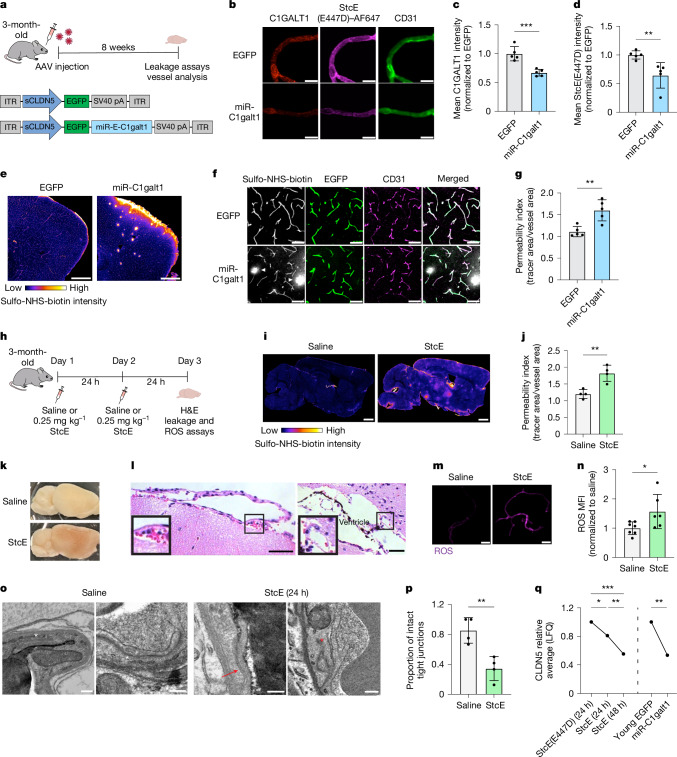
Reduced brain endothelial mucin-type O-glycosylation increases BBB leakiness and brain bleeding. **a**, Overview of AAV-mediated *C1galt1* knockdown paradigm. ITR, inverted terminal repeat. **b**, C1GALT1 expression and mucin-domain glycoprotein labelling in acutely isolated microvessels. Scale bars, 10 µm. **c**, Quantification of C1GALT1 expression in **b** (*n* = 5 mice per group, two-sided *t*-test; mean ± s.e.m.). **d**, Quantification of mucin-domain glycoprotein labelling in **b** (*n* = 5, two-sided *t*-test; mean ± s.e.m.). **e**, Sulfo-NHS-biotin leakage in the cortices of young mice transduced with AAV-EGFP and AAV-miR-C1galt1. Scale bars, 500 µm. **f**, Sulfo-NHS-biotin leakage (indicated by white arrowheads) from EGFP^+^ cortical vessels of AAV-miR-C1galt1-transduced mice. Scale bars, 50 µm. **g**, Quantification of vessel permeability in **f** (*n* = 5 mice per group; two-sided *t*-test; mean ± s.e.m.). **h**, Overview of luminal mucin-domain glycoprotein degradation paradigm using 48 h StcE treatment. H&E, haematoxylin and eosin. **i**, Whole-brain images of sulfo-NHS-biotin leakage in mice treated with StcE for 48 h. Leakage is indicated by light-coloured hotspots and higher overall signal throughout the brain. Scale bars, 1 mm. **j**, Quantification of cortical vessel permeability in **i** (*n* = 4 mice per group; two-sided *t*-test; mean ± s.e.m.). **k**, Brains from mice treated with StcE for 48 h exhibit haemorrhaging. **l**, H&E images of cerebral bleeds in the meninges and ventricles of mice treated with StcE for 48 h. Scale bars, 50 µm. **m**, ROS signal in acutely isolated microvessels from mice treated with StcE for 48 h. Scale bars, 25 µm. **n**, Quantification of ROS signal in **m** (*n* = 6–7 mice per group, two-sided *t*-test; mean ± s.e.m.). **o**, TEM of brain endothelial tight junctions showing intact tight junctions (white asterisk) and abnormal tight junctions (detached, red asterisk; discontinuity, red arrow) in mice treated for 24 h with saline or StcE. Scale bar, 200 nm. **p**, Quantification of intact tight junctions in mice treated for 24 h with saline or StcE (*n* = 4 mice per group; two-sided *t*-test; mean ± s.e.m.). **q**, Label-free quantification (LFQ) of CLDN5 in microvessels via mass spectrometry (*n* = 4–5 mice per group; two-sided *t*-test; mean ± s.e.m.).

**Extended Data Fig. 6:**
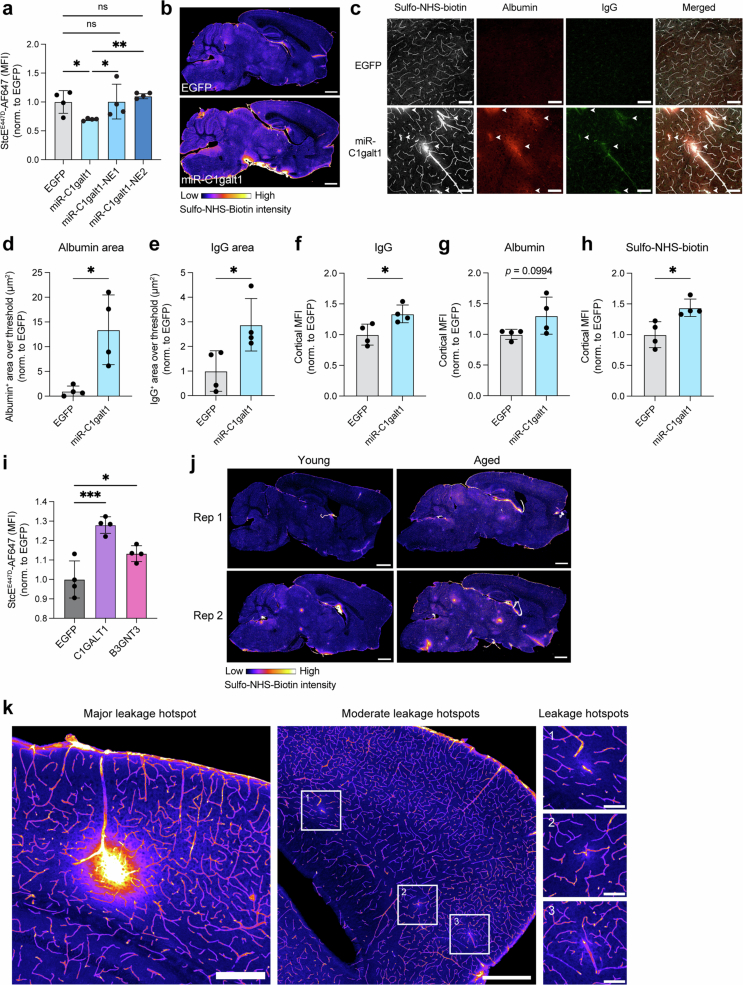
Additional characterization of brain endothelial mucin-type O-glycan biosynthetic enzyme knockdown and overexpression and BBB leakage. a) Reduction of StcE^E447D^-AF647 binding following knockdown of *C1galt1* in bEnd.3 cells using AAV-miR-E constructs compared to AAV-EGFP via flow cytometry (n = 4 per group; one-way ANOVA with the Šidák post hoc test; mean ± s.e.m.). b) Sulfo-NHS-biotin leakage in whole brain sections of AAV-transduced mice. Scale bar=1 mm. c) Sulfo-NHS-biotin, albumin, and IgG leakage (indicated by white triangles) from cortical vessels of AAV-transduced mice. Scale bar = 100 µm. d) Quantification of albumin signal area over background threshold in (c) (n = 4 mice per group; two-sided t-test; mean ± s.e.m.). e) Quantification of IgG signal area over background threshold in (c) (n = 4 mice per group; two-sided t-test; mean ± s.e.m.). f) Cortical IgG signal in (c) (n = 4 mice per group; two-sided t-test; mean ± s.e.m.). g) Cortical albumin signal in (c) (n = 4 mice per group; two-sided t-test; mean ± s.e.m.). h) Cortical sulfo-NHS-biotin signal in (c) (n = 4 mice per group; two-sided t-test; mean ± s.e.m.). i) Increased StcE^E447D^-AF647 binding following overexpression of C1GALT and B3GNT3 in bEnd.3 cells using AAV-C1GALT1 and AAV-B3GNT3 compared to AAV-EGFP via flow cytometry (n = 4 per group; one-way ANOVA with Dunnett’s post hoc test; mean ± s.e.m.). j) Whole brain images of sulfo-NHS-biotin leakage in young (3-month-old) and aged (23-month-old) wild-type C57BL/6 mice (n = 2 mice per group). Scale bar = 1 mm. k) High-resolution images of cortical sulfo-NHS-biotin leakage hotspots in aged AAV-EGFP-treated mouse brain image from Fig. 4g showing major and moderate hotspots of leakage. Scale bar = 250 µm (major leakage), 500 µm (moderate leakage), and 100 µm (three close-up images of moderate leakage).

To test the effects of downregulated mucin-type O-glycan biosynthesis on BBB function, we used a brain endothelial cell-specific adeno-associated virus (AAV) targeting approach to knock down *C1galt1* expression in mouse brain endothelial cells in vivo (Fig. 3a). C1GALT1 catalyses the addition of galactose to the core GalNAc-Ser/Thr residue to generate the core 1 O-glycan, a prominent mucin core structure on the endothelium^28,29^ (Fig. 2m). *C1galt1* was also selected for knockdown to better understand the effects of its downregulation in brain endothelial cells observed in both ageing and Alzheimer’s disease (Fig. 2a,b,k,l). Using the AAV9-derived capsid PHP.V1^30^ and an abbreviated *CLDN5* promoter (sCLDN5), we generated AAV-PHP.V1-sCLDN5::EGFP-miR-E-C1galt1 (hereafter referred to as AAV-miR-C1galt1) for brain endothelial cell-specific knockdown of *C1galt1*. We confirmed that AAV-miR-C1galt1 transfection of bEnd.3 cells decreased mucin-domain labelling via StcE(E447D)–AF647 compared with our control, AAV-PHP.V1-sCLDN5::EGFP (hereafter referred to as AAV-EGFP), and two less effective *C1galt1*-targeting miR-E constructs, AAV-miR-C1galt1-NE1 and AAV-miR-C1galt1-NE2 (Extended Data Fig. 6a and Supplementary Table 2). Eight weeks following intravenous injections of 3-month-old mice with AAV-miR-C1galt1 and AAV-EGFP, we detected robust EGFP expression in CD31^+^ brain endothelial cells (Fig. 3f). Furthermore, AAV-miR-C1galt1 effectively reduced brain endothelial C1GALT1 levels and StcE(E447D)–AF647 labelling compared with AAV-EGFP, notably to similar degrees as observed in aged mice compared with young mice (Figs. 1h,i, 2f,g and 3b–d). Using the small molecule tracer sulfo-NHS-biotin (0.5 kDa) to evaluate BBB permeability, we observed that mice transduced with AAV-miR-C1galt1 developed leaky BBBs with numerous hotspots of sulfo-NHS-biotin leakage outside of cortical blood vessels (Fig. 3e–g and Extended Data Fig. 6b). By contrast, the tracer remained confined in the BBB lumens of mice transduced with AAV-EGFP, suggesting that their BBBs remained intact and functional. Leakage of albumin and IgG were also observed in the cortices of mice treated with AAV-miR-C1galt1, often colocalizing with areas of sulfo-NHS-biotin leakage (Extended Data Fig. 6c–h). These results suggest that the brain endothelial downregulation of core 1 mucin-type O-glycan biosynthetic enzymes observed in ageing and neurodegenerative diseases may contribute to impaired BBB function in these states.

**Extended Data Fig. 7:**
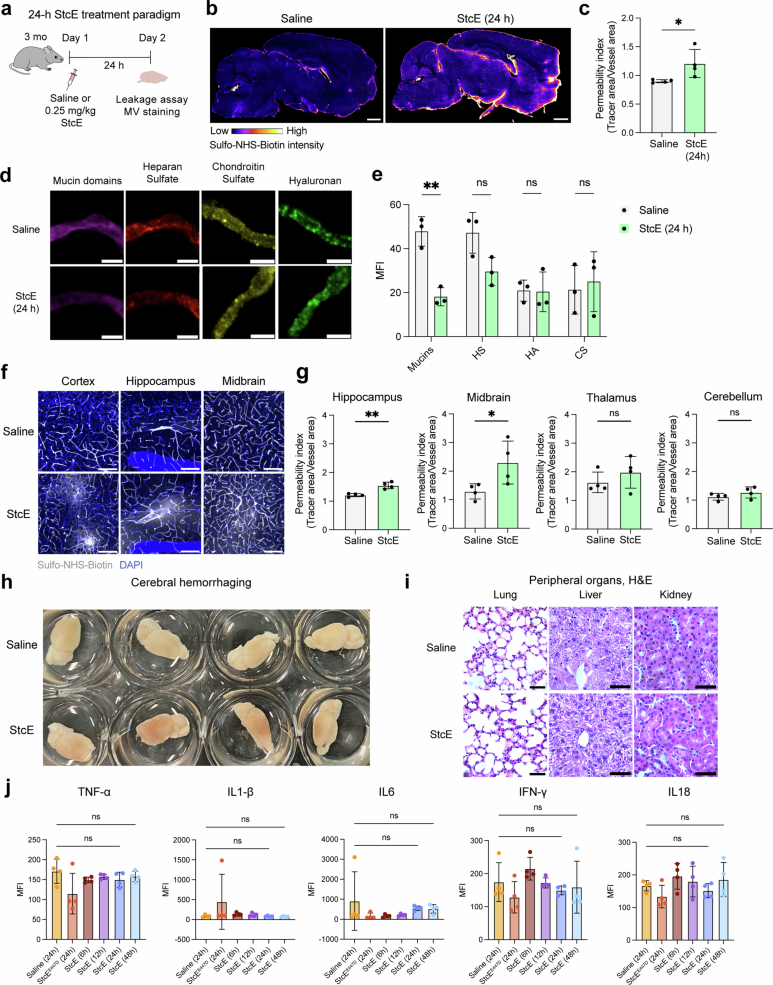
Additional characterization of StcE treatment in young mice. a) Overview of luminal mucin-domain glycoprotein degradation paradigm using 24-h StcE treatment. b) Whole brain images of sulfo-NHS-biotin leakage in StcE-treated mice at 24 h. Scale bar = 1 mm. c) Quantification of cortical vessel permeability to sulfo-NHS-biotin in (b) (n = 4 mice per group; two-sided t-test; mean ± s.e.m.). d) Mucin domain, heparan sulfate, chondroitin sulfate, and hyaluronan expression in acutely isolated microvessels from 24-h saline- and StcE-treated mice. Scale bar = 10 µm. e) Quantification of (d) (n = 3 mice per group, two-sided t-test; mean± s.e.m.). f) Sulfo-NHS-biotin leakage (white) from blood vessels in different brain regions of 48-h StcE-treated mice. Scale bar = 50 µm. g) Quantification of sulfo-NHS-biotin permeability in different brain regions in saline- and 48-h StcE-treated mice (n = 4 mice per group; two-sided t-test; mean ± s.e.m.). h) Additional brains from 48-h StcE-treated mice exhibiting hemorrhaging (n = 4 mice per group). i) H&E images of lung, liver, and kidney in saline and 48-h StcE-treated mice. Scale bar = 50 µm. j) Luminex quantification of major inflammatory cytokines in plasma from young mice following saline, StcE^E447D^, and StcE treatment. (n = 4 mice per group, one-way ANOVA with Dunnett’s post hoc test; mean ± s.e.m.).

To additionally test the effects of targeted disruption of luminal mucin-type O-glycosylation, we used intravenous injections of StcE to cleave luminal mucin-domain glycoproteins in vivo. We found that 24 h of StcE treatment in young mice led to significantly decreased StcE(E447D)–AF647 labelling and increased BBB permeability (Extended Data Fig. 7a–e). Furthermore, sustained two-day StcE treatment led to even greater widespread BBB dysfunction and unexpected cerebral haemorrhaging with noticeable red blood cell leakage in the meninges and ventricles (Fig. 3h–l and Extended Data Fig. 7f–h). Notably, we did not observe a cytokine storm in blood or haemorrhaging in peripheral tissues surveyed at this dose, indicating a lack of major systemic inflammatory response or global vascular disruption with StcE treatment (Extended Data Fig. 7i,j). A previous study found that *C1galt1*^−/−^ mice developed defective vascular networks and died in utero owing to CNS haemorrhaging^28^, further suggesting that the brain vasculature may be exceptionally sensitive to perturbations in mucin-type O-glycans.

**Extended Data Fig. 8:**
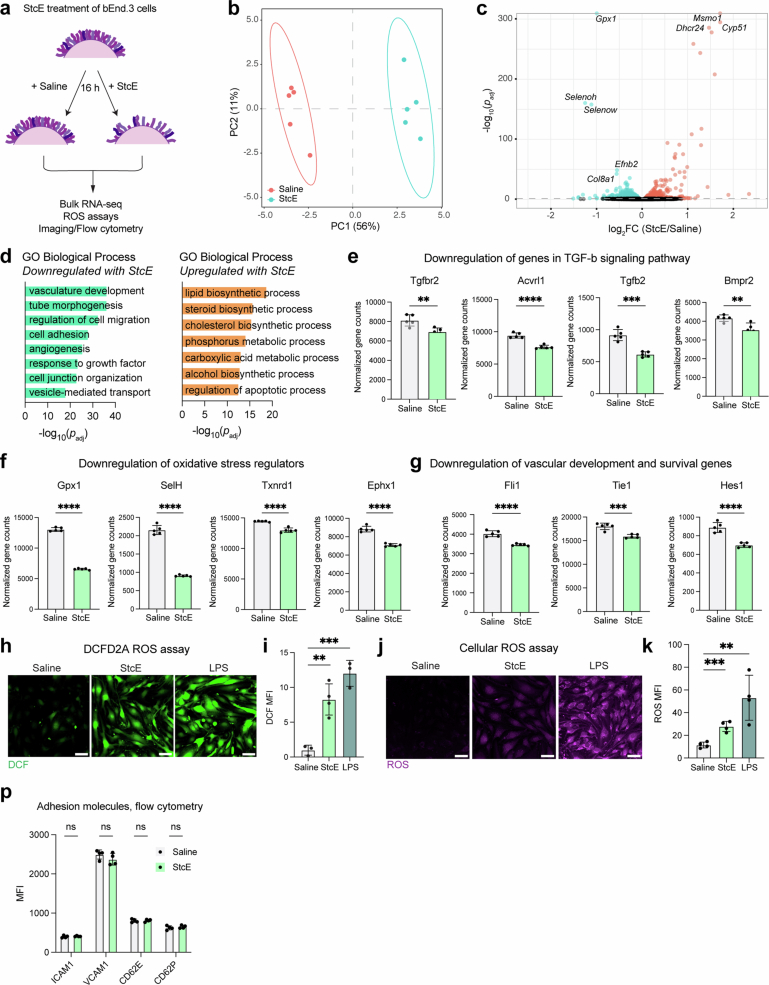
Mucin degradation downregulates vascular homeostatic genes and increases ROS generation in bEnd.3 cells. a) Overview of assays to characterize the effects of 16-h StcE treatment on bEnd.3 cells. b) PCA of bulk RNA-seq data of saline- and StcE-treated bEnd.3 cells (n = 5 wells per group). c) Volcano plot of gene expression changes induced by StcE treatment (genes upregulated by StcE treatment are in orange and genes downregulated by StcE treatment are in teal). d) Top upregulated and downregulated GO biological processes in bEnd.3 cells treated with StcE vs. saline. e) Normalized counts of genes involved in TGF-β signaling (n = 5 wells per group; two-sided t-test; mean ± s.e.m.). f) Normalized counts of genes involved in oxidative stress regulation (n = 5 wells per group; two-sided t-test; mean ± s.e.m.). g) Normalized counts of genes involved in vascular development and survival (n = 5 wells per group; two-sided t-test; mean ± s.e.m.). h) Immunofluorescence images of ROS signal in bEnd.3 cells as assayed by DCF fluorescence. Scale bar = 50 µm. i) Quantification of (h) (n = 3-4 wells per group; two-sided t-test; mean ± s.e.m.). j) Immunofluorescence images of ROS signal in bEnd.3 cells as assayed by the Cellular ROS Deep Red kit. Scale bar = 50 µm. k) Quantification of (j) (n = 3-4 wells per group; two-sided t-test; mean ± s.e.m.). l) Flow cytometry quantification of adhesion molecules in bEnd.3 cells treated with saline or StcE (n = 4 wells per group; two-sided t-test; mean ± s.e.m.).

**Extended Data Fig. 9:**
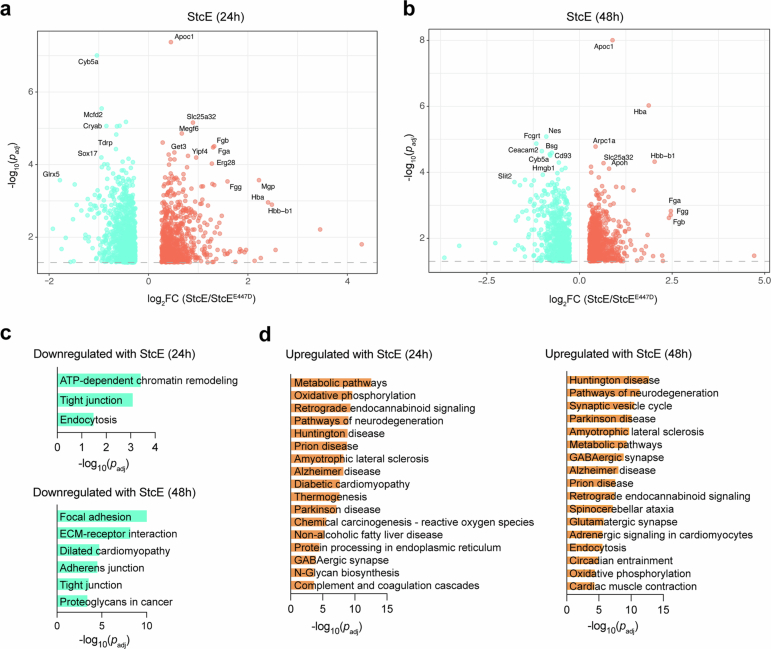
Cerebrovascular proteomics of StcE-treated mice. a) Volcano plot of cerebrovascular protein changes induced by 24-h StcE treatment (genes upregulated by StcE treatment are in orange and genes downregulated by StcE treatment are in teal). b) Volcano plot of cerebrovascular protein changes induced by 48-h StcE treatment (genes upregulated by StcE treatment are in orange and genes downregulated by StcE treatment are in teal). c) Top downregulated KEGG pathways with StcE treatment. d) Top upregulated KEGG pathways with StcE treatment.

To better understand the molecular changes in brain endothelial cells induced by mucin degradation, we performed bulk RNA-seq on bEnd.3 cells after 16 h of StcE treatment (Extended Data Fig. 8a,b). Differential gene expression analysis revealed significant downregulation of genes involved in vascular development and integrity (*Fli1*, *Tie1* and *Hes1*), TGFβ signalling (*Tgfbr2*, *Acvrl1* and *Tgfb2*), and oxidative stress regulation (*Gpx1*, *SelH* and *Txnrd1*) (Extended Data Fig. 8c–g). Given that the most downregulated genes included scavengers of reactive oxygen species (ROS), we conducted cellular ROS assays and found increased ROS production in StcE-treated bEnd.3 cells and in brain microvessels acutely isolated from StcE-treated mice (Fig. 3m,n and Extended Data Fig. 8h–k). Increased oxidative stress can damage macromolecules and has been linked to BBB breakdown in ageing and CNS diseases^7,9,31^. Ultrastructural analysis of the BBB revealed a higher frequency of abnormal, discontinuous tight junctions in StcE-treated mice compared with saline-treated controls (Fig. 3o,p). Congruently, mass spectrometry analysis identified a decrease in tight junction proteins, including CLDN5, following StcE treatment at 24 h, with an even greater reduction at 48 h, and similarly following *C1galt1* knockdown (Fig. 3q and Extended Data Fig. 9a–d). These findings highlight the critical role of mucin-domain glycoproteins in preserving brain endothelial homeostasis and BBB integrity.

## Glycocalyx restoration improves brain homeostasis

**Fig. 4:**
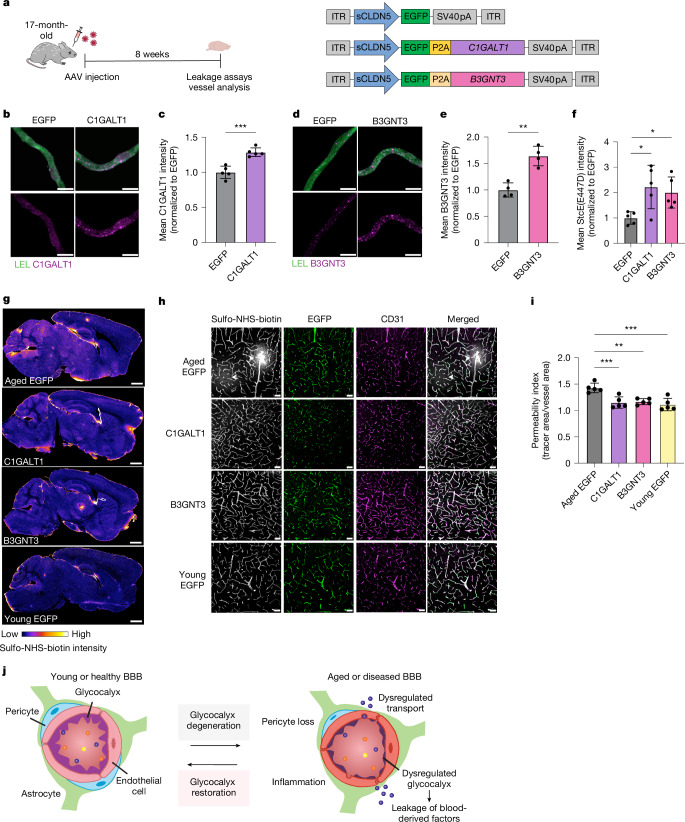
Restoration of mucin-type O-glycosylation improves BBB function in aged mice. **a**, Overview of C1GALT1 and B3GNT3 overexpression paradigm and relevant AAV constructs. **b**, C1GALT1 expression in acutely isolated microvessels labelled with LEL. Scale bars, 10 µm. **c**, Quantification of **b** (*n* = 5 mice per group; two-sided *t*-test; mean ± s.e.m.). **d**, B3GNT3 expression in acutely isolated microvessels labelled with LEL. Scale bar = 10 µm. **e**, Quantification of **d** (*n* = 5 mice per group; one-way ANOVA with Dunnett’s post hoc test; mean ± s.e.m.). **f**, Quantification of mucin-domain glycoprotein labelling in acutely isolated microvessels (*n* = 5 mice per group; two-sided *t*-test; mean ± s.e.m.). **g**, Sulfo-NHS-biotin leakage in whole-brain sections of AAV-transduced mice. **h**, Sulfo-NHS-biotin leakage (indicated by arrowheads) from EGFP^+^ cortical vessels of AAV-transduced mice. Scale bars, 50 µm. **i**, Quantification of **h** (*n* = 5 mice per group; one-way ANOVA with Dunnett’s post hoc test; mean ± s.e.m.). **j**, Schematic of BBB dysfunction during ageing and neurodegenerative disease, highlighting new findings from this paper. The brain endothelial glycocalyx layer degenerates with ageing, thereby contributing to dysregulated BBB function. Restoring the glycocalyx may be an effective therapeutic approach for recovering BBB function in ageing-associated disease conditions.

We next tested whether we could reverse the detrimental effects of downregulated brain endothelial mucin-type O-glycosylation in aged mice by overexpressing two age-downregulated mucin-type O-glycan biosynthetic enzymes, C1GALT1 and B3GNT3, in brain endothelial cells (Figs. 2f–i and 4a). Transfection of bEnd.3 cells with AAV-PHP.V1-sCLDN5::EGFP-C1GALT1 and AAV-PHP.V1-sCLDN5::EGFP-B3GNT3 (hereafter referred to as AAV-C1GALT1 and AAV-B3GNT3, respectively) plasmids increased mucin-domain labelling via StcE(E447D)–AF647 compared with transfection with AAV-EGFP (Extended Data Fig. 6i). After 8 weeks post-AAV injection of 17-month-old mice, we verified robust EGFP expression in CD31^+^ brain endothelial cells with all constructs and increased brain endothelial cell levels of C1GALT1 and B3GNT3 with transduction of AAV-C1GALT1 and AAV-B3GNT3, respectively, compared with AAV-EGFP (Fig. 4b–e,h). We also observed increased StcE(E447D)–AF647 cerebrovascular labelling in mice transduced with AAV-C1GALT1 and AAV-B3GNT3 compared with AAV-EGFP (Fig. 4f). Upon evaluation of BBB integrity in these mice, we found that aged mice transduced with AAV-EGFP exhibited heterogeneous sulfo-NHS-biotin leakiness broadly across brain regions, confirming expected signs of BBB dysfunction in aged mice (Fig. 4g and Extended Data Fig. 6j,k). However, aged mice transduced with AAV-C1GALT1 and AAV-B3GNT3 exhibited significantly reduced sulfo-NHS-biotin leakage from blood vessels broadly across the brain, comparable to levels observed in young mice (Fig. 4g–i). These data demonstrate that increasing core 1 mucin-type O-glycosylation on the brain endothelium can effectively improve BBB function in aged mice (Fig. 4j).

**Fig. 5:**
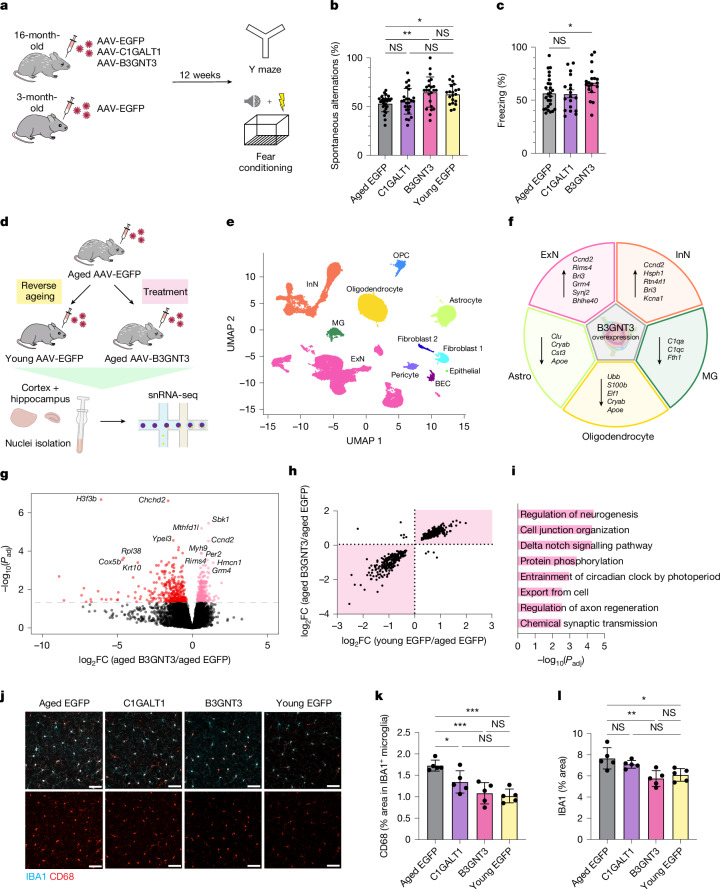
Restoration of mucin-type O-glycosylation increases neuronal homeostasis, reduces glial inflammation and improves memory and learning in aged mice. **a**, Experimental scheme used for behavioural testing of mice. **b**, Spatial working memory assessment using the Y maze (*n* = 26 (aged EGFP), 19 (C1GALT1), 23 (B3GNT3) and 20 (young EGFP); one-way ANOVA with Tukey’s post hoc test; mean ± s.e.m.). **c**, Hippocampal-dependent learning and memory assessment by contextual fear conditioning (*n* = 26 (EGFP), 18 (C1GALT1) and 19 (B3GNT3); one-way ANOVA with Dunnett’s post hoc test; mean ± s.e.m.). **d**, Outline of snRNA-seq profiling of pooled cortical and hippocampal tissue of young AAV-EGFP, aged AAV-EGFP and aged AAV-B3GNT3-treated groups. **e**, Uniform manifold approximation and projection (UMAP) of 69,250 nuclei from pooled cortical and hippocampal tissue of young AAV-EGFP, aged AAV-EGFP and aged AAV-B3GNT3-treated groups (*n* = 3 mice per group), coloured by cell type. BEC, brain endothelial cell; ExN, excitatory neuron; InN, inhibitory neuron; MG, microglia; OPC, oligodendrocyte precursor cell. **f**, Overview of key DEGs in each major cell type induced by AAV-B3GNT3 treatment in aged mice compared with AAV-EGFP. Astro, astrocyte. **g**, Volcano plot of ExN DEGs induced by AAV-B3GNT3 treatment in aged mice (upregulated genes in pink and downregulated genes in red). *P*_adj_, adjusted *P* value. **h**, Comparison of ExN DEG fold changes with B3GNT3 overexpression (*y* axis) and reverse ageing (*x* axis). Areas in which AAV-B3GNT3 treatment causes changes in the reverse direction of ageing are highlighted. **i**, Top upregulated pathways in ExN based on DEGs shared between AAV-B3GNT3 treatment and reverse ageing (top right quadrant in **h**). **j**, IBA1 and CD68 expression in the cortices of AAV-transduced mice. Scale bars, 50 µm. **k**, Quantification of CD68^+^ signal in IBA1^+^ microglia in **j** (*n* = 5 mice per group; one-way ANOVA with Tukey’s post hoc test; mean ± s.e.m.). **l**, Quantification of cortical IBA1^+^ area in **j** (*n* = 5 mice per group; one-way ANOVA with Tukey’s post hoc test; mean ± s.e.m.).

As leakage of blood-derived molecules into the brain has previously been shown to promote a more inflammatory and neurotoxic brain environment^7,32^, we tested whether increasing core 1 mucin-type O-glycosylation and, consequently, reducing BBB leakiness, could promote homeostatic changes in brain state and function. To assess changes in cognitive function, we subjected the three AAV-treated aged mice groups to memory and learning tasks using the Y maze and fear conditioning tests 12 weeks post-AAV injection (Fig. 5a). Remarkably, aged mice treated with AAV-B3GNT3 exhibited significantly increased spontaneous alternations in the Y maze and increased contextual freezing, indicative of improvements in spatial working memory and hippocampal-dependent learning and memory, respectively (Fig. 5b,c). Aged mice treated with AAV-C1GALT1 did not exhibit significant improvements in either behavioural test, and no significant difference was observed in cued freezing among the three aged groups.

**Extended Data Fig. 10:**
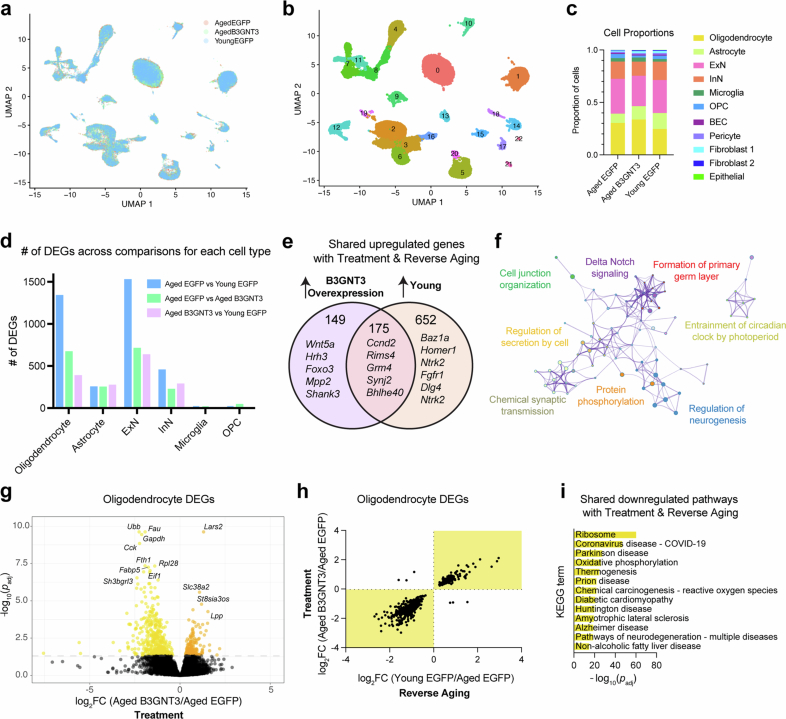
Additional snRNA-seq analysis. a) UMAP of 69,250 nuclei from pooled cortical and hippocampal tissues of young AAV-EGFP, aged AAV-EGFP, and aged AAV-B3GNT3 groups (n = 3 mice per group), colored by experimental group. b) UMAP of 69,250 nuclei from the cortex and hippocampus of young AAV-EGFP, aged AAV-EGFP, and aged AAV-B3GNT3 groups (n = 3 mice per group), colored by cluster. c) Summary quantification of the proportion of captured cell types by treatment group. d) Summary of the number of DEGs across comparisons of aged AAV-EGFP, aged AAV-B3GNT3, and young AAV-EGFP for each major cell type. e) Venn diagram of the number and examples of excitatory neuronal DEGs upregulated with AAV-B3GNT3 treatment in aged mice and with reverse aging. f) Network analysis of shared excitatory neuronal pathways upregulated with aged AAV-B3GNT3 and in reverse aging. g) Volcano plot of DEGs in oligodendrocytes induced by AAV-B3GNT3 treatment in aged mice (genes upregulated with AAV-B3GNT3 treatment in orange and genes downregulated with AAV-B3GNT3 treatment in yellow). h) Comparison of log_2_-transformed fold changes of DEGs with B3GNT3 overexpression (Aged B3GNT3/Aged EGFP) and reverse aging (Young EGFP/Aged EGFP). Areas in which AAV-B3GNT3 treatment causes changes in the opposite direction of aging are highlighted. i) Top downregulated pathways in oligodendrocytes based on DEGs shared between AAV-B3GNT3 treatment and reverse aging (bottom left quadrant in (h)).

To characterize the cellular and molecular changes in the aged brain induced by brain endothelial B3GNT3 overexpression, we performed snRNA-seq on pooled cortical and hippocampal tissues isolated from aged AAV-EGFP, aged AAV-B3GNT3 and young AAV-EGFP treatment groups (Fig. 5d). This yielded 69,250 single-nucleus transcriptomes with representation from all major brain cell types (Fig. 5e and Extended Data Fig. 10a,b). Although no significant changes in cell-type proportions were observed among the three groups (Extended Data Fig. 10c), pseudobulk analysis revealed gene expression changes across all major cell types, with the largest number of differentially expressed genes (DEGs) observed in excitatory neurons and oligodendrocytes (Extended Data Fig. 10d). Further interrogation of these two cell populations revealed that many DEGs induced by AAV-B3GNT3 occurred in the opposite direction to those observed with ageing, suggesting that overexpression of brain endothelial B3GNT3 promotes cellular changes towards a more youthful brain state (Fig. 5g–i and Extended Data Fig. 10e–i). Indeed, aged mice treated with AAV-B3GNT3 exhibited increased expression of genes related to neuronal homeostasis (*Rims4*, *Wnt5a* and *Foxo3*) and neurogenesis (*Smad1*, *Syngap1* and *Ccnd2*) in neuronal populations and reduced expression of activation- and disease-associated genes in glial cell populations including astrocytes (*Clu*, *Cryab* and *Apoe*) and microglia (*C1qa* and *C1qc*) (Fig. 5f–i and Extended Data Fig. 10e–i). Additional immunofluorescence characterization of neuroinflammatory markers revealed a significant reduction in the microglial activation marker CD68 in aged mice treated with AAV-B3GNT3 and, to a lesser extent, AAV-C1GALT1 (Fig. 5j–l). These results demonstrate that brain endothelial B3GNT3 overexpression promotes the restoration of homeostatic brain processes that correlate with improved cognitive outcomes in aged mice.

## Discussion

In summary, we identify brain endothelial glycocalyx dysregulation as a molecular mechanism that contributes to BBB dysfunction in ageing and neurodegenerative diseases. We observe a conserved decline in brain endothelial mucin-type O-glycosylation in these conditions and demonstrate that reduction of this modification leads to increased BBB permeability and susceptibility to cerebral bleeding. AAV-mediated overexpression of brain endothelial mucin-type O-glycan biosynthetic enzymes restored BBB integrity, reduced markers of neuroinflammation and improved cognitive function in aged mice. These results demonstrate that restoring the brain endothelial glycocalyx may be an effective therapeutic route to combat BBB breakdown in age-related CNS diseases.

Although glycosaminoglycans have been widely studied as major constituents of the glycocalyx^5^, our work highlights important contributions of mucin-domain glycoproteins to the brain endothelial glycocalyx layer and BBB function. There remains much to be learned about the specific mechanisms by which mucin-domain glycoproteins affect BBB function. The glycocalyx forms a physical and charge barrier on the vascular lumen, and removal of any major component, including glycosaminoglycans, as shown in previous studies, or mucin-domain glycoproteins, as shown in this work, seems to destabilize the glycocalyx layer and increase vascular permeability^3,33^. In addition to contributing to the structural integrity of the glycocalyx layer, mucin-domain glycoproteins have diverse biological roles, including in signalling, cell–cell interactions and regulation of membrane morphology^6,20–22^. Their removal from brain endothelial cells broadly compromises BBB integrity, including by modulating tight junctions, increasing oxidative stress and disrupting other crucial vascular homeostatic pathways. Further studies will be essential to identify the key mucin-domain glycoproteins involved in BBB regulation and the specific molecular pathways through which they act.

Although our study shows that AAV-mediated overexpression of C1GALT1 and B3GNT3 reduces BBB permeability and improves brain health, the precise mechanisms that underlie these beneficial effects remain unclear. By limiting the nonspecific uptake of blood-derived molecules, these treatments may help protect the brain from harmful circulatory factors, such as albumin, IgG and fibrinogen, which have been shown to trigger neuroinflammatory changes in the brain^7,32^. However, it is important to note that C1GALT1 and B3GNT3 are likely to influence a wide range of proteins and glycan structures. Understanding the molecular pathways affected by these glycosyltransferases will be crucial for our understanding of brain ageing and rejuvenation.

Finally, although this work focuses on the contributions of mucin-domain glycoproteins to BBB function, we found that many additional brain endothelial glycosylation pathways were dysregulated in ageing and neurodegenerative diseases (Fig. 1e,f and Extended Data Fig. 5a–f). Future work investigating the roles of the diverse glycans and glycoconjugates that constitute the brain endothelial glycocalyx layer will be fundamental to our understanding of brain function and neurodegeneration.

## Methods

### Animals

Aged C57BL/6 mice (16–21 months old) were obtained from the National Institute on Aging rodent colony. Young C57BL/6 mice (3 months old) were obtained from Jackson Laboratories. All experiments used male mice. All mice were kept on a 12 h–12 h light–dark cycle and provided ad libitum access to food and water. All animal care and procedures complied with the Animal Welfare Act and were in accordance with institutional guidelines and approved by the Veterans Affairs Palo Alto Committee on Animal Research and the institutional administrative panel of laboratory animal care at Stanford University.

### Human tissue

Post mortem fresh-frozen brain tissues were obtained from Stanford/VA Aging Clinical Research Center with approval from the Stanford Institutional Review Board and patient consent. Autopsies were performed no more than 12 h after death, and all samples used in this study were stored at −80 °C until the time of processing. Group characteristics are summarized in Supplementary Data 2. Individuals in the Alzheimer’s disease group were both clinically diagnosed and pathologically determined to exhibit Alzheimer’s disease brain hallmarks including β-amyloid and tau pathophysiology.

### Transmission electron microscopy

Transmission electron micrographs of the brain endothelial glycocalyx layer were obtained as described^4^ with some modifications. In brief, young (3-month-old) and aged (21-month-old) mice were perfused with ice-cold fixation buffer composed of 2% glutaraldehyde (EMS), 2% sucrose, 0.1 M sodium cacodylate buffer (EMS), and 2% lanthanum nitrate (EMS) through the left ventricle using a peristaltic pump at 2 ml min^−1^. The brain was removed and sliced coronally using a matrix into 1-mm-thick sections. Cortical punches (1 mm^3^) were cut and immersed in perfusion solution for 2 h before storage overnight in perfusion solution without glutaraldehyde at 0 °C. The following day, the samples were washed in a 0.03 M NaOH 2% sucrose solution and subjected to an ascending ethanol gradient and embedded in epoxy resin (EMS). 90 nm sections were cut using a Leica UC6 ultramicrotome (Leica Microsystems) and collected onto formvar-coated 50-mesh copper grids. The grids were post-stained with 2% uranyl acetate followed by Reynold’s lead citrate for 5 min each. Sections were imaged using a Tecnai 12 120 kV TEM (FEI), and data were recorded using either an UltraScan 1000 with Digital Micrograph 3 software (Gatan) or a Rio16 CMOS camera with GWS software (Gatan). Between 6 and 12 cortical capillaries per animal were captured by a blinded observer. Quantitative analysis of endothelial glycocalyx thickness and area were performed using ImageJ software (Extended Data Fig. 1a). For StcE treatment experiments, 3-month-old mice were retro-orbitally injected with 0.25 mg kg^−1^ StcE or inactivated StcE(E447D) 24 h before perfusion.

For BBB ultrastructural analysis, mice were injected retro-orbitally with 0.3 ml of 0.5 mg g^−1^ of HRP type II in PBS (Sigma, P8250). After 30 min, brains were dissected and fixed in 0.1 M sodium cacodylate buffer (EMS) with 5% glutaraldehyde (EMS) and 4% PFA (EMS) for 1 h at room temperature and then for 16 h in 4% PFA/0.1 M cacodylate at 4 °C. Tissues were washed overnight with 0.1 M sodium cacodylate at 4 °C and then sliced coronally using a matrix into 1-mm-thick sections. Cortical punches (0.5–1 mm^3^) were cut and incubated in 0.5 mg ml^−1^ of 3,3′-diaminobenzidine with 0.01% hydrogen peroxide in TBS for 45 min at room temperature. Tissues were washed with TBS overnight, post-fixed in 2% osmium tetroxide and 2.5% potassium ferrocyanide in 0.1 M sodium cacodylate, and en bloc stained with 1% uranyl acetate and Walton’s lead aspartate stain. Samples were then dehydrated in an ascending ethanol gradient and embedded in epoxy resin (EMS). Eighty-nanometre sections were cut using a Leica UC7 ultramicrotome (Leica Microsystems) and collected on formvar-coated 100-mesh copper grids. The grids were post-stained with 3.5% uranyl acetate followed by Sato’s lead citrate. Sections were imaged using a Tecnai 12 120 kV TEM (FEI), and data were recorded using a Rio16 CMOS camera with GWS software (Gatan). The assessment of tight junctions in the images was performed in a blinded manner.

### Flow cytometry on acutely isolated brain endothelial cells

Microvessels from young (3-month-old) and aged (21-month-old) mice were isolated as previously described with some modifications^26,34^. In brief, mice were euthanized via CO_2_, and brains were retrieved in PBS supplemented with 1% bovine serum albumin (BSA) and 1× cOmplete protease inhibitor cocktail (Millipore Sigma) on ice. The olfactory bulb was discarded, and meningeal vessels were removed by gentle rolling on blotting paper. Brains were minced using a razor blade on ice and then homogenized using a loose-fit, 7 ml Dounce (Wheaton) in 1% BSA-PBS with 1× protease inhibitor. The homogenate was centrifuged in 27% (wt/vol) 70 kDa dextran (Sigma) in HBSS at 4,400*g* for 25 min. Myelin and parenchymal cell layers were removed. Pelleted microvessels were deposited on a pre-wet 40-μm strainer, washed with PBS, and mechanically dissociated into single cells as previously described^26^. For enzymatic dissociation of brain tissue, previously published protocols were used^12,13^. Pelleted cells were suspended in FACS buffer (1% BSA in PBS) and stained on ice for 30 min with the following antibodies: rat anti-CD31-PE/CF594 (1:100, BD, 563616), rat anti-CD45-PE/Cy7 (1:200, Biolegend, 103114), mouse anti-heparan sulfate (1:100, Amsbio, clone 10E4, 370255-1), mouse anti-chondroitin sulfate (1:100, Sigma, clone CD-56, C8035), biotinylated HABP (1:150, Amsbio, AMS.HKD-BC41), fluorescein-conjugated SNA (1:300, Vector Labs, FL-1301-2), biotinylated MAAII (1:300, Vector Labs, B-1265-1), and StcE(E447D)–AF647 (5 μg ml^−1^). After washing cells with FACS buffer, secondary incubation with Alexa Fluor-conjugated streptavidin (1:1,000, Thermo Fisher Scientific) and secondary antibodies (1:400, Thermo Fisher Scientific) was carried out on ice for 20 min. Live cells were identified using Sytox Blue viability dye (1:1,000, Thermo Fisher Scientific, S34857). Flow cytometry analysis was performed on a BD LSRFortessa, and data were analysed using FlowJo software (TreeStar).

### Microvessel imaging

Cerebral microvessels were isolated for immunofluorescence imaging using the same protocol described above for flow cytometry, except, instead of dissociating microvessels, they were fixed on 40-μm strainers with 4% PFA in PBS at room temperature for 15 min with gentle rocking. Microvessels were then washed with PBS and mounted on poly-d-lysine-coated slides (Thermo Fisher Scientific). Microvessels were blocked in 3% normal donkey serum (Jackson ImmunoResearch) with 0.3% Triton X-100 (Sigma) in TBS-T (1× TBS with 0.05% Tween-20) for 1 h, followed by 1 h incubation at room temperature with primary antibodies. For glyco-profiling of cerebral microvessels, the same primary antibodies, lectins, binding proteins and concentrations from our flow panel were used along with fluorescein-conjugated VVA (1:300, Vector Labs, FL-1231-2). Additional primary antibodies used include rabbit anti-C1GALT1 (1:100, Thermo Fisher Scientific, PA5-52814), rabbit anti-B3GNT3 (1:100, Thermo Fisher Scientific, PA5-21988), rabbit anti-CAV1 (1:100, Cell Signaling Technologies; 3267S), goat anti-CD31 (1:100, R&D, AF3628) and fluorescein-conjugated LEL (1:250, Vector Labs, FL-1171-1). For secondary staining, microvessels were washed three times with TBS-T for 5 min each, followed by incubation with Alexa Fluor-conjugated antibodies (1:250, Thermo Fisher Scientific) or streptavidin (1:1,000, Thermo Fisher Scientific) for 1 h at room temperature. Microvessels were then washed three times again and coverslipped with Vectashield Hardset Antifade Mounting Medium with DAPI (Vector Labs, H-1500-10) or ProLong Gold Antifade Mountant (Thermo Fisher Scientific, P36934). Imaging was performed on a confocal laser-scanning microscope (Zeiss LSM880). All observed single-plane microvessels were captured and then quantified using ImageJ software.

### Immunofluorescence analysis

For luminal vascular labelling, mice were euthanized with 2.5% (v/v) Avertin and transcardially perfused via peristaltic pump at 2 ml min^−1^ with the following ice-cold solutions: 8 ml PBS, 10 ml of 5 μg ml^−1^ of StcE(E447D)–AF647 or SNA–Cy3 (Vector Labs, CL-1303-1), and 8 ml of 4% PFA. For all other immunofluorescence analysis, mice were euthanized with 2.5% (v/v) Avertin and manually perfused with PBS unless noted otherwise. Tissues were extracted and fixed in 4% PFA at 4 °C overnight before preservation in 30% sucrose in PBS. Tissues were sectioned into 40 μm slices using a microtome (Leica). Slices were subsequently blocked in 3% normal donkey serum with 0.3% Triton X-100 in TBS-T for 1.5 h at room temperature and incubated at 4 °C overnight with the following primary antibodies: goat anti-CD31 (1:100, R&D, AF3628), goat anti-Iba1 (1:100, Abcam, ab5076), rat anti-CD68 (1:100, Bio-Rad, MCA1957), goat anti-collagen type IV (1:100, Sigma, AB769), rat anti-NID1 (1:100, Thermo Fisher Scientific, MA1-06501), rabbit anti-CLDN5 (1:100, Thermo Fisher Scientific, 34–1600), mouse anti-ZO1 (1:100, Thermo Fisher Scientific, 33–9100), goat anti-albumin (1:100, Thermo Fisher Scientific, A90-134A), goat anti-PODXL (1:100, R&D Systems, AF1556). The following day, slices were washed three times with TBS-T, stained with the appropriate Alexa Fluor-conjugated secondary antibodies (1:250, Thermo Fisher Scientific) or Alexa Fluor-conjugated streptavidin (1:1,000, Thermo Fisher Scientific) for 2 h at room temperature, washed three times again, mounted, and coverslipped with Vectashield Hardset Antifade Mounting Medium with DAPI (Vector Labs, H-1500-10). Imaging was performed on a confocal laser-scanning microscope (Zeiss LSM880), and images were analysed using ImageJ. Luminal vascular coverage was calculated as vessel (CD31^+^ or COL4A^+^) area occupied by the marker of interest divided by total vessel area. Endothelial MFI calculated using CD31^+^ mask.

### BBB leakage assay

Mice were anaesthetized and injected retro-orbitally with Sulfo-NHS-biotin (Thermo Fisher Scientific, 21335) at 0.25 mg g^−1^ body weight. The tracer was allowed to circulate for 5 min before perfusion with PBS. Hemibrains were post-fixed in 4% PFA overnight at 4 °C, cryopreserved in 30% sucrose, and sagittally sectioned into 40-μm slices. Sections were blocked and co-stained with CD31 and the appropriate secondary antibody as described earlier and Alexa Fluor 647-conjugated streptavidin (1:1,000, Thermo Fisher Scientific). Images were taken on a confocal laser-scanning microscope (Zeiss LSM880) and analysed using ImageJ software. Multicoloured gradient images were generated using the fire LUT in ImageJ. Permeability index of vessels was determined as the area occupied by tracer divided by the vessel area.

### In vivo StcE treatment and cerebral bleeding assessment

All recombinant StcE proteins were produced as described^22,23^. For all applications, proteins were run through Pierce high-capacity endotoxin removal columns (Thermo Fisher Scientific) at least seven times following manufacturer’s instructions. Endotoxin levels were tested using HEK-Blue lipopolysaccharide (LPS) Detection Kit 2 (InvivoGen) according to manufacturer recommendations. Mice were injected retro-orbitally with 0.25 mg kg^−1^ StcE once a day for 2 days before perfusion with ice-cold PBS. Cerebral bleeding was visualized by eye post-perfusion and by H&E staining. For H&E staining, hemibrains and peripheral organs were formalin-fixed and paraffin embedded (FFPE) and cut into 5-μm-thick sagittal sections mounted on slides. Sections were deparaffinized in xylene (3 times, 3 min), hydrated in a series of graded alcohols (2× 100%, 1× 95%, 1× 80%; 3 min each), and stained with Richard Allan haematoxylin (4 min; followed by 30 s in 4% acetic acid in water and dipping in 0.3% ammonia water) and eosin with phloxine (30 s). Sections were then dehydrated (10 dips in 95% ethanol followed by 2× 1 min in 100% ethanol), cleared in xylene (3 times, 1 min), and coverslipped prior to imaging on a wide-field microscope (Zeiss AxioImager).

### Luminex cytokine measurement

Plasma cytokine measurement was performed using the Luminex assay at the Human Immune Monitoring Center at Stanford University. The mouse 48-plex Procarta kit (Thermo, EPX480-20834-901) was used according to the manufacturer’s instructions. Plasma samples were diluted 1:3 and run in singlet on a 96-well plate alongside standard curve and quality control samples. Custom Assay Chex control beads (Radix BioSolutions) were added to all wells to assess nonspecific binding.

### Luminal cerebrovascular proteome enrichment and peptide preparation

Young (3-month-old) and aged (21-month-old) mice (*n* = 6 mice per group) were euthanized with 2.5% (v/v) Avertin and transcardially perfused via peristaltic pump with the following ice-cold solutions: 8 ml of PBS, 20 ml of 0.5 mg ml^−1^ Sulfo-NHS-biotin (Thermo Fisher Scientific, 21217), and 10 ml of 50 mM Tris-PBS. Cerebral microvessels were isolated as described in earlier sections and collected for lysis via sonication in RIPA buffer (Thermo Fisher Scientific) supplemented with 1× protease inhibitor. Lysates were centrifuged at 13,000*g* for 15 min at 4 °C, and supernatant was saved for downstream enrichment. Protein concentration was measured by BCA (Pierce), and 1 mg of each sample was incubated with 70 μl of streptavidin magnetic beads (Thermo Fisher Scientific, 88817) at 4 °C overnight. The following day, beads were processed for proteomic analysis using a published protocol with some modificiations^35^. In brief, beads were washed once with 200 μl of 50 mM Tris-HCl (pH 7.5) and twice with 200 μl of 2 M urea in 50 mM Tris (pH 7.5) and incubated in 80 μl 2 M urea in 50 mM Tris with 1 mM dithiothreitol (Thermo Fisher Scientific, R0861) and 0.4 μg trypsin/LysC (Fisher, V5073) at 25 °C for 1 h shaking at 1,000 rpm. Supernatant was further reduced with 4 mM dithiothreitol for 30 min with shaking and alkylated with 10 mM iodoacetamide (IAA, Sigma, I1149) for 45 min in the dark with shaking. An additional 0.5 μg of trypsin/LysC was added to samples for overnight digestion at 37 °C with shaking. The following day, samples were acidified to 1% vol/vol formic acid and desalted using BioPureSPN mini C18 columns (Nest Group). All centrifugation steps were carried out at 50*g* for 1 min at room temperature. Columns were washed with 200 μl HPLC-grade methanol (Fisher) and equilibrated two times with 200 μl 0.1% formic acid in HPLC-grade water (Fisher) (solvent A). Samples were loaded onto columns, washed 4 times with solvent A, and eluted 2 times with 75 μl 0.1% formic acid in 80% acetonitrile (solvent B). The combined elutes were dried in a vacuum concentrator and reconstituted in 10 μl solvent A for LC–MS/MS.

### Luminal cerebrovascular mass spectrometry-based proteomics

LC–MS/MS analysis was performed on a Q Exactive HF-X (Thermo Fisher Scientific) with an UltiMate 3000 RSLCnano system (Thermo Fisher Scientific). Peptides were loaded on an in-house 75-μm (inner diameter) capillary column packed with 40 cm of ReproSil-Pur 120 C18-AQ 1.9 μm resin (Dr. Maisch). Chromatographic separation was achieved using a flow rate of 300 nl min^−1^ with the following 120 min gradient: 96% A + 4% B for 18 min, 70% A + 30% B for 72 min, 60% A + 40% B for 15 min, and 4% A + 96% B for 15 min, where solvent A was 0.1 % formic acid in HPLC-grade water (Fisher) and solvent B was 0.1% formic acid in HPLC-grade acetonitrile (Fisher). Full MS scans were acquired at a resolution of 60,000, with an automatic gain control (AGC) target of 3 × 10^6^, maximum injection time (IT) of 20 ms, and scan range 300–1,650 *m*/*z* in a data-dependent mode. MS2 scans were acquired with the following parameters: resolution of 15,000, AGC target of 1 × 10^5^, maximum IT of 54 ms, loop count 15, TopN 15, isolation window 1.4 *m*/*z*, fixed first mass 100.0 *m*/*z*, normalized collision energy (NCE) 28 units, charge exclusion of unassigned, 1, 6–8 and >8, peptide match preferred, exclude isotopes on, and fragmented *m*/*z* values were dynamically excluded from further selection for a period of 45 s. Raw data were processed and analysed using MaxQuant and Perseus^36^. In brief, peptide spectral matches were made against a target-decoy *Mus musculus* reference proteome database downloaded from Uniprot. Methionine oxidation and N-terminal acetylation were specified as variable modifications, and carbamidomethylation of cysteines was specified as a fixed modification. Precursor ion search tolerance of 20 ppm and product ion mass tolerance of 20 ppm were used for searches. Both unique and razor peptides were used for quantification. Results were filtered to a 1% false discovery rate (FDR) at the peptide and protein levels. Proteins were quantified and normalized using MaxLFQ^37^ with a LFQ minimum ratio count set to 1. For quantitative comparative analysis, protein intensity values were log2-transformed, and missing values were imputed from a normal distribution with width 0.3 and downshift value of 1.8 using Perseus. Principal component analysis (PCA) was performed in Perseus using the Benjamini–Hochberg FDR with a cutoff of 0.05. GO term enrichments were performed using DAVID^38^ with the *M. musculus* proteome as a background.

### Microvessel mass spectrometry-based proteomics

Brain microvessels were isolated and lysed in 1× RIPA buffer with 1× cOmplete protease inhibitor cocktail (Sigma) on ice. Lysates were centrifuged at 13,000*g* for 15 min at 4 °C, and supernatant protein concentration was measured by microBCA (Pierce). Samples were then reduced and alkylated by TCEP and CAA, followed by protein purification using SP3 (Cytiva). Samples were digested with 200 ng trypsin/LysC in pH 7.8 at 37 °C for 3 h, desalted, and eluted following the manufacturer’s instructions. Samples were acidified to 0.1% formic acid and filtered before loading onto the nanoLC system. One microgram protein was injected for all samples. LC–MS/MS analysis was performed on the TimsTOF Pro (Bruker Daltonics) coupled with the NanoElute system (Bruker Daltonics) with solvent A (0.1% formic acid in water) and solvent B (0.1% formic acid in acetonitrile). Tryptic peptides were loaded first on the trapping column Waters ACQUITY UPLC M-Class Symmetry C18 Trap Column, 100 A, 5 μm, 180 μm × 20 mm, and eluted with analytical column, IonOpticks Aurora Elite CSI 15 × 75 C18 UHPLC column. Elution gradient was set as 0 min 5% B; 9 min 12% B; 9.1 min 12% B; 27 min 30% B; 27.5 min 85% B and 36 min 85% B with a flow rate of 0.35 μl min^−1^ from 0 min to 9 min and reduced to 0.3 μl min^−1^ at 9.1 min until the end of the gradient. Eluted peptides were measured in diaPASEF mode with base method *m*/*z* range 100–1,700 and 1/k0 range of 0.85–1.30 V s cm^−2^. The source parameters were 1,400 V for capillary voltage, 3.0 l min^−1^ for dry gas, and 180 °C for dry temperature using Captive Spray (Bruker Daltonics). Collision energies (27 eV and 45 eV) were allocated for 1/K0 = 0.85 V s cm^−2^ and 1/K0 = 1.30 V s cm^−2^, respectively. Data were processed using Spectronaut (Biognosys AG, v19.1) for directDIA search with Swiss-Prot Mouse database downloaded on 3 March 2023. Default settings were used with a slight modification of minimum peptide length 6. Candidates were filtered using *Q* < 0.05 and absolute average log_2_ ratio ≥0.263.

### RNA-seq glycosylation-related gene analysis

Previously published ageing and neurodegenerative disease RNA-seq datasets demonstrating robust brain endothelial cell enrichment were chosen for glycosylation-related gene analysis^12,26,27^. We filtered for glycosylation-related genes based on KEGG (Kyoto Encyclopedia of Genes and Genomes) listed glycosylation enzymes and related proteins. Most glycoproteins were excluded due to the enormous variety of members in this family which possess biological functions not directly relevant to glycosylation. Significantly upregulated and downregulated glycosylation-related genes in each dataset were used for Reactome pathway analysis with *P*_adj_ < 0.05 set as the threshold for significant enrichment.

### Bulk RNA-seq on bEnd.3 cells

The mouse brain endothelial cell line bEnd.3 (ATCC, CRL-2299) was cultured in high-glucose DMEM (Thermo Fisher Scientific, 10567022) supplemented with 10% fetal bovine serum (FBS) and 1% penicillin/streptomycin and maintained in a humidified incubator containing 5% CO_2_ at 37 °C. For bulk RNA-seq analysis, bEnd.3 cells were grown in 6-well plates and treated with 5 nM StcE for 16 h at 37 °C. Cells were lysed and collected into RNAse-free Eppendorf tubes for total RNA extraction using the RNeasy Plus Micro kit (Qiagen, 74034). RNA quantity and quality were assessed by an Agilent 2100 Bioanalyzer (Agilent Technologies). All samples passed a high quality control threshold (RNA integrity number ≥9.7) and proceeded to cDNA library preparation by Novogene. Libraries were sequenced on the NovaSeq 6000 (paired-end, 2× 150 bp depth). Trimmed reads were aligned to the *M. musculus* reference genome GRCm38. Differential gene expression analysis and visualization were performed using DESeq2 (v1.32) (Supplementary Data 5). Genes with a *P*_adj_ < 0.05 were used for GO biological pathway enrichment analysis.

### bEnd.3 immunofluorescence analysis

For confocal imaging analysis, bEnd.3 cells were plated on round coverslips (EMS, 72196-12) in a 24-well plate and treated with 5 nM StcE for 16 h at 37 °C. Cells were fixed in 4% PFA for 15 min, blocked in 3% normal donkey serum with 0.3% Triton X-100 in PBS for 1 h at room temperature, and incubated at room temperature in blocking solution with the following primary antibodies for 1.5 h: goat anti-CD31 (1:100, R&D, AF3628), mouse anti-ZO1 (1:100, Thermo Fisher Scientific, 33–9100), rabbit anti-CAV1 (1:100, Cell Signaling Technologies; 3267S) and mouse anti-CLTC (1:100, Thermo Fisher Scientific, MA1-065). Cells were subsequently washed 3 times with PBS, stained with the appropriate Alexa Fluor-conjugated secondary antibodies (1:250, Thermo Fisher Scientific) for 1 h at room temperature, washed 3 times again, mounted, and coverslipped with Vectashield Hardset Antifade Mounting Medium with DAPI (Vector Labs, H-1500-10) or ProLong Gold Antifade Mountant (Thermo Fisher Scientific, P36934). Imaging was performed on a confocal laser-scanning microscope (Zeiss LSM880), and images were analysed using ImageJ.

For flow cytometry analysis, bEnd.3 cells were plated in a 24-well plate and treated with 5 nM StcE for 16 h at 37 °C. Cells were collected from plates using enzyme-free cell dissociation buffer (Thermo Fisher Scientific, 1315014) and resuspended in 1% BSA in PBS (FACS buffer). Cells were stained with the following antibodies on ice in FACS buffer for 30 min: APC-anti-CD54 (1:100, BioLegend, 116120), FITC-anti-VCAM1 (1:100, Thermo Fisher Scientific, 11-1061-82), Alexa Fluor 647-anti-CD62P (1:100, BD Biosciences, 563674), anti-CD62E (1:100, Thermo Fisher Scientific, 14-0627-82) followed by Alexa Fluor 555-conjugated anti-mouse IgG (1:400, Thermo Fisher Scientific). Live cells were identified using Sytox Blue viability dye (1:1,000, Thermo Fisher Scientific, S34857). Flow cytometry analysis was performed on a Sony SH800S sorter, and data were analysed using FlowJo software (TreeStar). Only live, singlet cells were used for analysis of adhesion molecule MFI.

### ROS assays

For in vitro ROS assays, bEnd.3 cells were plated in 8-well µ-slides (Ibidi, 80826) and treated with 5 nM StcE, 5 μg ml^−1^ of LPS, or an equivalent volume of saline in complete medium for 16 h at 37 °C. Cells were then washed two times with PBS, and cellular ROS assays were carried out according to manufacturer instructions. In brief, 20 µM of DCFDA (Abcam, ab113851) and 1× ROS Deep Red Dye (Abcam, ab186029) were used for their respective assays and incubated with cells for 30 min at 37 °C in the dark. Cells were washed three times with PBS and imaged immediately on a confocal laser-scanning microscope (Zeiss LSM880). Quantification of MFI was performed using ImageJ software. For in vivo ROS assays, cerebral microvessel were isolated as described earlier and incubated with 1× ROS Deep Red Dye (Abcam, ab186029) for 30 min at 37 °C in the dark. Microvessels were then washed with 5 ml of PBS and pelleted by centrifugation at 2,000*g* for 10 min and transferred to 8-well µ-slides (Ibidi, 80826) for immediate imaging on a confocal laser-scanning microscope (Zeiss LSM880). All observed single-plane microvessels were captured and then quantified using ImageJ software.

### Western blot

Whole-brain microvessel lysates (30 µg) were boiled in 1× NuPAGE LDS Sample Buffer (Thermo Fisher Scientific) and 5% 2-mercaptoethanol (Sigma) at 95 °C for 10 min. Lysates and the Precision Plus Protein Dual Color Standards (Bio-Rad, 1610374) were loaded onto a 4–12% NuPAGE Bis-Tris precast gel (Thermo Fisher Scientific) and run in NuPAGE MOPS buffer (Thermo Fisher Scientific) at 160 V for 1 h. The gel was transferred to a 0.45-µm nitrocellulose membrane (Bio-Rad) using the Mini Trans-Blot Cell (Bio-Rad) and NuPAGE transfer buffer (Thermo Fisher Scientific) at 100 V for 1.25 h. Protein transfer was verified using Ponceau S staining for 5 min followed by de-staining in de-ionized water. The membrane was blocked with 5% non-fat dry milk (Bio-Rad) in TBS-T for 1 h at room temperature and then incubated with IRDye 800CW streptavidin (1:2,500, LI-COR Biosciences) in 5% non-fat dry milk (Bio-Rad) in TBS-T for 1 h at room temperature. The membrane was then washed three times with TBS-T for 5 min each and imaged on the LI-COR Odyssey DLx.

### Design and cloning of pAAV-sCLDN5 constructs

The Ple261 MiniPromoter (‘sCLDN5’) pEMS1938 was a gift from E. Simpson (Addgene plasmid #82563). A *cis* rAAV genome plasmid with AAV2 inverted terminal repeats was utilized for cloning of a sCLDN5 and EGFP reporter using restriction enzymes and In-Fusion Snap Assembly (Takara Bio). To knock down *C1galt1* in brain endothelial cells, de novo predictions of small interfering RNA (siRNA) guides targeting *C1galt1* were generated using the DSIR algorithm^39^ and subsequently filtered using ‘Sensor rules’ to select for sequences with highly favourable small hairpin RNA (shRNA) features^40,41^. Three de novo 97-mer miR-E shRNA sequences (Supplementary Table 2) were synthesized (IDT) and inserted into pAAV-sCLDN5-EGFP using restriction enzyme cloning for in vitro evaluation. To overexpress C1GALT1 and B3GNT3 in brain endothelial cells, P2A-C1GALT1 and P2A-B3GNT3 were cloned into pAAV-sCLDN5-EGFP using restriction enzyme cloning to generate pAAV-sCLDN5-EGFP-P2A-C1GALT1 and pAAV-sCLDN5-EGFP-P2A-B3GNT3, respectively.

### In vitro pAAV evaluation

bEnd.3 cells were split into a 24-well plate 1 day before transfection. Cells at approximately 70% confluency were transfected with the pAAVs described in the previous section via Lipofectamine 3000 according to the manufacturer’s recommendations (Thermo Fisher Scientific, L3000008). After 16 h, the cell medium was replaced with fresh, pre-warmed medium. Then, 2.5 days after transfection, cells were collected from plates using enzyme-free cell dissociation buffer (Thermo Fisher Scientific, 1315014) and resuspended in FACS buffer. Cells were stained with StcE(E447D)–AF647 (5 μg ml^−1^) on ice for 20 min. Live cells were identified using Sytox Blue viability dye (1:1,000, Thermo Fisher Scientific, S34857). Flow cytometry analysis was performed on a Sony SH800S sorter, and data were analysed using FlowJo software (TreeStar). Only live, singlet, EGFP^+^ cells were used for analysis of StcE(E447D)–AF647 MFI.

### AAV production

For production of PHP.V1-sCLDN5::EGFP and all other plasmids containing the PHP.V1 (pUCmini-iCAP-PHP.V1 was a gift from V. Gradinaru; Addgene plasmid #127847) capsid, AAV production was performed in-house utilizing a previously published protocol^42^. In brief, triple transfection of HEK293T cells was performed on 90–95% confluent cells in DMEM containing Glutamax supplemented with 5% FBS and non-essential amino acids. Fresh, warm medium was replaced 12 h post-transfection. Medium was collected 72 h post-transfection. Fresh, warm medium was added and collected along with cells 120 h post-transfection and combined with the previous fraction. Cells and medium were centrifuged at 2,000*g* for 15 min at room temperature. Supernatant was collected in a separate bottle and combined with 40% (wt/vol) PEG (final concentration 8% wt/vol PEG) and incubated on ice for 2 h before transferring to 4 °C overnight. Cell pellet was resuspended in buffer containing salt-active nuclease (SAN, ArcticZymes Technologies) and incubated at 37 °C for 1 h before transferring to 4 °C overnight. PEG medium was centrifuged at 4,000*g* for 30 min at 4 °C. After centrifugation, supernatant was bleached and discarded. PEG pellet was resuspended in SAN + SAN buffer, combined with the previous fraction, and incubated at 37 °C for an additional 30 min. Lysate was centrifuged at 2,000*g* for 15 min at room temperature, and supernatant was loaded onto an iodixanol gradient (15%, 25%, 40% and 60% fractions). Gradients were transferred to an ultracentrifuge (Beckman Coulter) using a Type 70 Ti rotor set at 350,000*g* for 2 h and 25 min at 18 °C. AAV particles were collected from the 40/60% interface, washed in PBS, and concentrated using an Amicon Ultra-15 (Millipore Sigma) filter device with a 100 kDa cutoff. AAV titration was performed using the AAVpro Titration Kit (for Real Time PCR) Ver.2 (Takara Bio). AAVs were injected retro-orbitally at 8 × 10^11^ viral genomes per mouse.

### snRNA-seq of mouse brain tissue

Pooled cortical and hippocampal tissue were dissected from frozen brain hemispheres from young (5-month-old) and aged (19-month-old) mice that had been injected with AAVs 8 weeks prior to takedown. Brain tissue was minced using a razor blade on ice and transferred to 2 ml glass Dounce tissue grinders (Sigma, D8938) with 2 ml EZ lysis buffer (Sigma, NUC-101) on ice. Tissues were homogenized using 25 strokes with pestle A followed by 25 strokes with pestle B, incorporating a 180° twist. Tissue homogenate was incubated on ice for 5 min and then centrifuged at 500*g* for 5 min at 4 °C. The pellet was resuspended with 4 ml EZ lysis buffer, incubated on ice for another 5 min, and pelleted as before. The pellet was then resuspended with chilled PBS and filtered through a 35-µm strainer before blocking with Mouse Fc block (1:50, BD 553142) in FACS buffer for 5 min. Nuclei were subsequently stained with AF647-anti-NeuN (1:100, Abcam, ab190565) in FACS buffer with 0.2 U µl^−1^ RNase inhibitor (Takara, 2313 A) for 30 min on ice, washed with FACS buffer, and resuspended in FACS buffer with Hoechst dye (1:2,000, Thermo Fisher, H3570) and 0.2 U µl^−1^ RNase inhibitor (Takara, 2313 A). Using a Sony MA900 Cell Sorter, approximately 50,000 singlet nuclei were sorted into 1.5 ml Eppendorf tubes containing 10 mg ml^−1^ UltraPure BSA (Thermo Fisher, AM2618) and 0.2 U µl^−1^ RNase inhibitor (Takara, 2313 A) in PBS. Collected nuclei were centrifuged at 400*g* for 5 min at 4 °C with slow deceleration. Supernatant was removed leaving about 40 µl of suspended nuclei. Nuclei were counted using a haemocytometer (Sigma, Z359629) and assessed for concentration and quality. snRNA-seq libraries were prepared using the Chromium Single Cell 3′ Kit v3.1 (10X Genomics) according to the manufacturer’s protocols, targeting 10,000 nuclei per sample. Twelve polymerase chain reaction (PCR) cycles were applied to generate cDNA, and eleven PCR cycles were applied for final library generation. Quality control of cDNA and libraries were conducted using a High Sensitivity D5000 ScreenTape (Agilent). The final snRNA-seq libraries were sequenced on a NovaSeq X.

### snRNA-seq quality control

Gene counts were obtained by aligning reads to the *M. musculus* reference genome GRCm38 using CellRanger software (v4.0.0) (10X Genomics). Ambient RNA was removed from each sample using SoupX (v1.6.2) and droplets containing multiple nuclei were filtered out using DoubletFinder (v2.0.4). We used Seurat (v4.1.1) to further exclude cells with fewer than 200 or more than 5,000 features and cells with more than 10% mitochondrial genes. In total, 69,250 nuclei remained and were used for further analysis (Supplementary Fig. 3a–d).

### Normalization and cell clustering

Each sample was normalized using Seurat’s SCTransform function, and samples were integrated using Seurat’s anchoring and integration functions. PCA was then run on the integrated dataset and the first fifteen principal components were used to generate a shared-nearest-neighbours graph. Clustering of nuclei was determined using the Louvain algorithm with a resolution of 0.3. UMAP was performed using the first 15 principal components and 30 nearest neighbours. Clusters were annotated for cell type using common cell-type markers identified by the FindConservedMarkers function applied to each cluster (Supplementary Fig. 4a; Supplementary Data 4).

### Pseudobulk differential gene expression

Pseudobulk counts were derived by aggregating raw counts for each sample using Seurat’s AggregateExpression function. DESeq2 was then used to perform bulk data normalization and differential gene expression across groups using default parameters. *P* value correction was carried out using the Benjamini–Hochberg procedure (FDR = 0.05) for each comparison. Genes with FDR <0.05 were used for subsequent KEGG pathway enrichment and Metascape^43^ analysis (Supplementary Data 5).

### Behavioural assays

In the Y maze test, the Y maze is made up of 3 white, opaque plastic arms at 120° angles from each other. At the beginning of the trials, mice were placed in the end of 1 arm and allowed to freely explore all 3 arms for 5 min. An arm entry was defined as having all four limbs inside an arm. The maze was cleaned with 70% ethanol between animals and before the first animal to eliminate traces of odour. The number of arm entries and triads (a set of consecutive arm entries) were recorded. The spontaneous alternation was calculated by dividing the number of triads by the number of possible alternations multiplied by 100.

In fear conditioning tests, mice were trained to associate cage context and an audiovisual cue with an aversive stimulus (foot shock). On day 1 (training), mice were placed in a cage and exposed to two periods of 30 s of paired cue light and 1,000-Hz tone followed by a 2-s foot shock (0.6 mA), with a 180-s interval. On day 2 (contextual fear conditioning), mice were re-exposed to the same cage context and freezing behaviour was recorded during minutes 1.5 to 6 using a FreezeScan tracking system (Cleversys). On day 4 (cued fear conditioning), mice were placed in a novel context that contained different odour, floor texture, and chamber walls and re-exposed the same cue light and tone from day 1 after 2 min of exploration. Freezing behaviour was recorded for 1.5–6 min following the cue using the FreezeScan tracking system (Cleversys).

### Statistical analysis

Data handling and statistical analyses were performed using R studio (v4.1.1) and GraphPad Prism Software unless stated otherwise. All statistical analyses comparing measurements between two groups were carried out using unpaired two-tailed Student’s *t*-tests. All statistical analyses comparing measurements between three or more groups were carried out using one-way ANOVA tests with post hoc tests for multiple comparisons. A *P* value of <0.05 was considered significant. All experimental procedures involving multiple experimental groups were carried out in an alternating fashion when possible to, avoid temporal and technical biases. Data in Figs. 1b–d,h–o, 2a,b,f–j, 3e–n, 4f–i and 5j–l and Extended Data Figs. 2a, 3f–i, 7a–c,h,i and 8h–k were successfully replicated in at least two independent experiments.

### Reporting summary

Further information on research design is available in the Nature Portfolio Reporting Summary linked to this article.

## Online content

Any methods, additional references, Nature Portfolio reporting summaries, source data, extended data, supplementary information, acknowledgements, peer review information; details of author contributions and competing interests; and statements of data and code availability are available at 10.1038/s41586-025-08589-9.

## Supplementary information

Supplementary Information

Reporting Summary

Supplementary Data 1 Young and aged luminal cerebrovascular proteomics.

Supplementary Data 2 Human samples information.

Supplementary Data 3 Bulk RNA-seq DEGs.

Supplementary Data 4 snRNA-seq cell-type markers.

Supplementary Data 5 snRNA-seq DEGs.

## Data Availability

Raw mass spectrometry data generated in this study have been deposited in the PRIDE database^44^ under accession codes PXD043964 and PXD058690. Raw sequencing data have been deposited in the NCBI Gene Expression Omnibus (GEO) under accession codes GSE268263 and GSE268642.
