# Supplementary material for: Glycocalyx dysregulation impairs blood–brain barrier in ageing and disease
Source: Nature. 2025 Feb 26;639(8056):985–94. doi: 10.1038/s41586-025-08589-9 (PMC11946907; doi:10.1038/s41586-025-08589-9)
Supplement: Supplementary file 1 — Supplementary Information [file 41586_2025_8589_MOESM1_ESM.docx]

**Supplementary Notes**

*Relative sizing of illustrated glycocalyx components*

Although Fig. 1a is not drawn to scale for readability purposes, the approximate sizes of glycoconjugates were considered in the relative sizing of glycocalyx components (smaller vs. larger). Precise structural information about glycosylated species is rare because glycans are flexible and not easily characterized using canonical structural biology techniques. Thus, relative size estimates of different glycocalyx components were approximated using the following assumptions and calculations:

- *Proteoglycans* – Proteoglycan core proteins (HSPGs, CSPGs) average around 20-45 kDa^1^, likely only reaching up to ~10 nm from the surface. Heparan sulfate and chondroitin sulfate molecules are typically 20-200 disaccharide repeats long. Each disaccharide unit is about 0.4 nm, which yields approximately 8-80 nm in length. This yields potential total distances of these proteins to reach up to about 100 nm.
- *Hyaluronan* – Hyaluronan has been shown to have an average molecular weight of 200kDa in the mouse brain^2^. 200kDa divided by 401.3Da per disaccharide = 498 disaccharide units. Each disaccharide unit is about 0.4 nm, which yields approximately 200 nm in length. Of course, there are much larger hyaluronan chains that will have an even longer predicted length and will form the longest glycan species in the glycocalyx layer.
- *Glycoproteins* – Glycoproteins vary greatly in size. A very large glycoprotein like LRP1 (600 kDa) is estimated to be around 60 nm long^3^, but most other glycoproteins are shorter.
  - *Mucin-domain glycoproteins* – Mucin domains are known to form extended bottlebrush structures that extend away from the cell membrane due to steric repulsions between the densely packed, highly negatively charged glycans, enabling greater length per amino acid than most other protein classes. A recent *Science* paper conducted single-molecule imaging on different glycoconjugate species and estimated the size of a 148 amino acid MUC1 mucin domain to be ~50nm^4^. Using this approximation, for a large mucin-domain glycoprotein like HEG1 which contains a predicted mucin domain of 1049 amino acids, this would yield ~350nm in predicted length not including the additional amino acid sequence outside of the mucin domain. For a smaller mucin-domain glycoprotein like PODXL which has a predicted mucin domain of about 310 amino acids, this approximation would yield ~100nm in predicted length.
- *Glycolipids* – Glycolipids are the shortest class of glycoconjugates, composed of monosaccharide or oligosaccharide groups attached to lipids anchored to the membrane.

From these approximations, the lengths of the different glycocalyx components depicted in Fig.1a from longest to shortest are: hyaluronan > mucin-domain glycoproteins ≥ proteoglycans > other glycoproteins > glycolipids.

**Supplementary Table 1. Average normalized RNA counts, total MS intensity, and total MS/MS counts of canonical brain endothelial cell (BEC) proteins, proteoglycans, and mucin-domain glycoproteins expressed in BECs.**


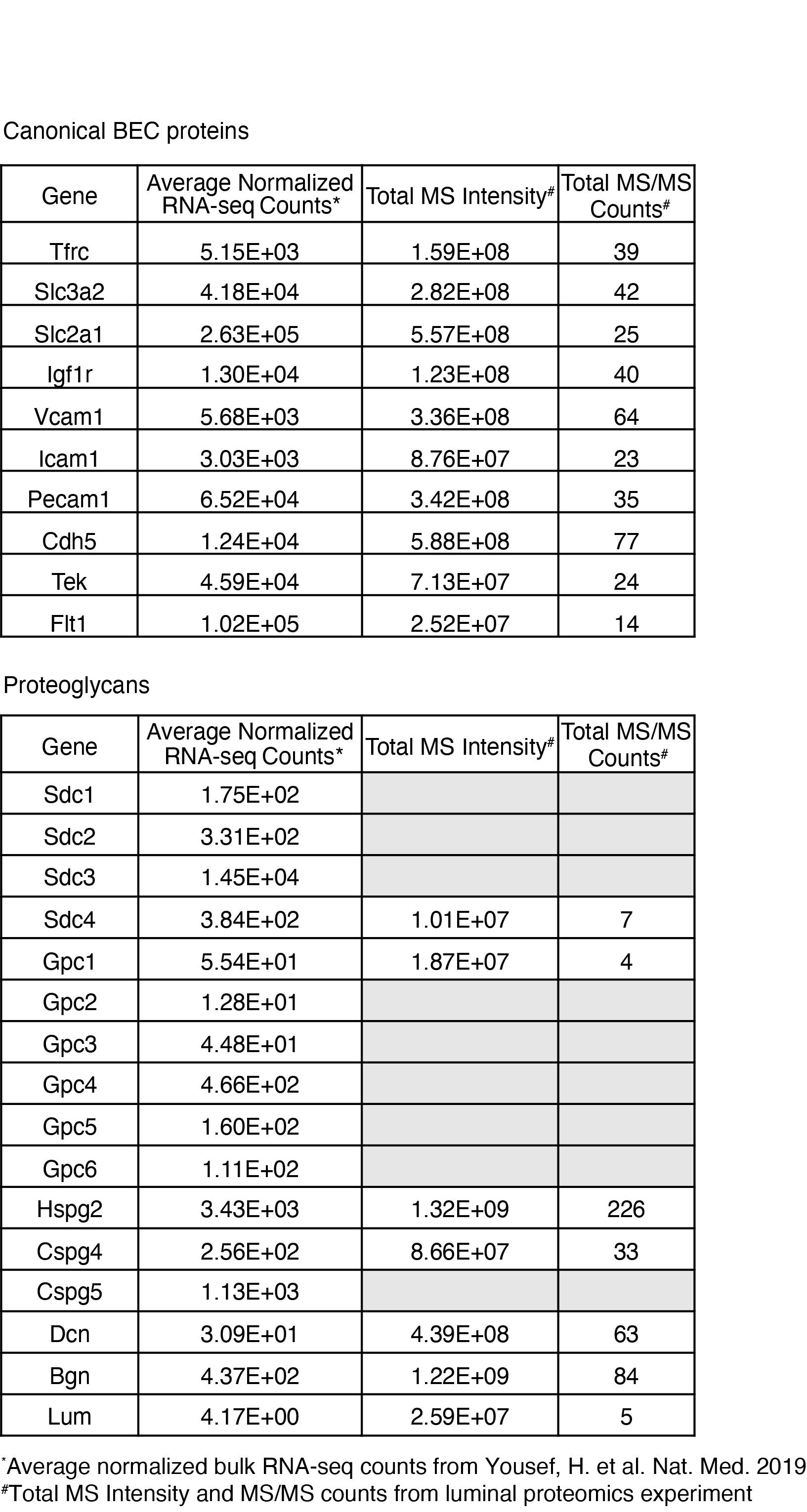


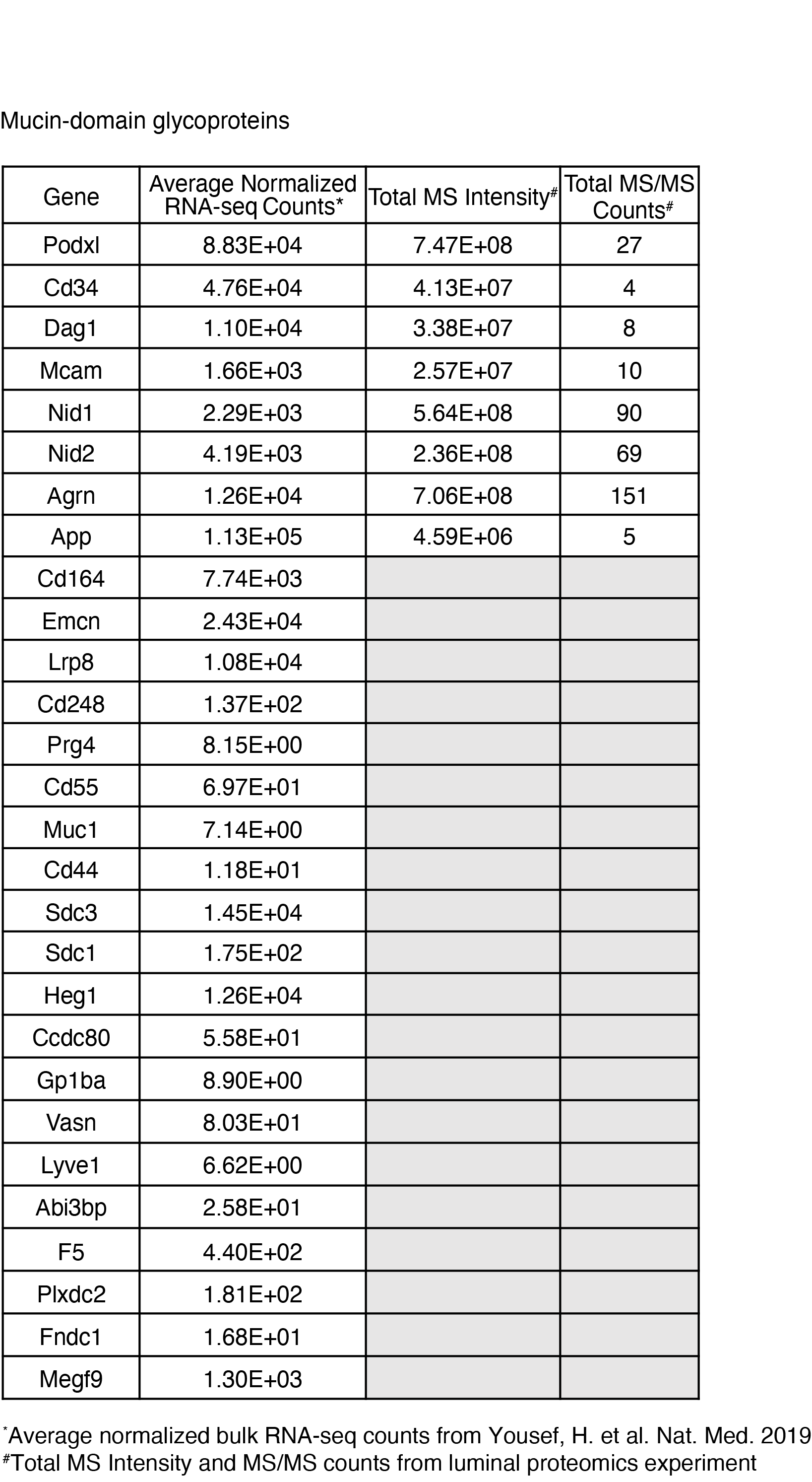


**Supplementary Table 2. miR-E constructs tested for the knockdown of *C1galt1***

| Plasmid Name | 22-nt guide | 97-mer shRNA sequences |
| --- | --- | --- |
| AAV-miR-E-C1galt1 | TCAGCGTTGTAATAAAGTGTTA | TGCTGTTGACAGTGAGCGTCAGCGTTGTAATAAAGTGTTATAGTGAAGCCACAGATGTATAACACTTTATTACAACGCTGGTGCCTACTGCCTCGGA |
| AAV-miR-E-C1galt1-NE1 | GACACCACTTAATCAAAGGTTA | TGCTGTTGACAGTGAGCGGACACCACTTAATCAAAGGTTATAGTGAAGCCACAGATGTATAACCTTTGATTAAGTGGTGTTTGCCTACTGCCTCGGA |
| AAV-miR-E-C1galt1-NE2 | TCAACATAAAGATGAGAACATA | TGCTGTTGACAGTGAGCGTCAACATAAAGATGAGAACATATAGTGAAGCCACAGATGTATATGTTCTCATCTTTATGTTGGTGCCTACTGCCTCGGA |

**Supplementary Figure 1**

**
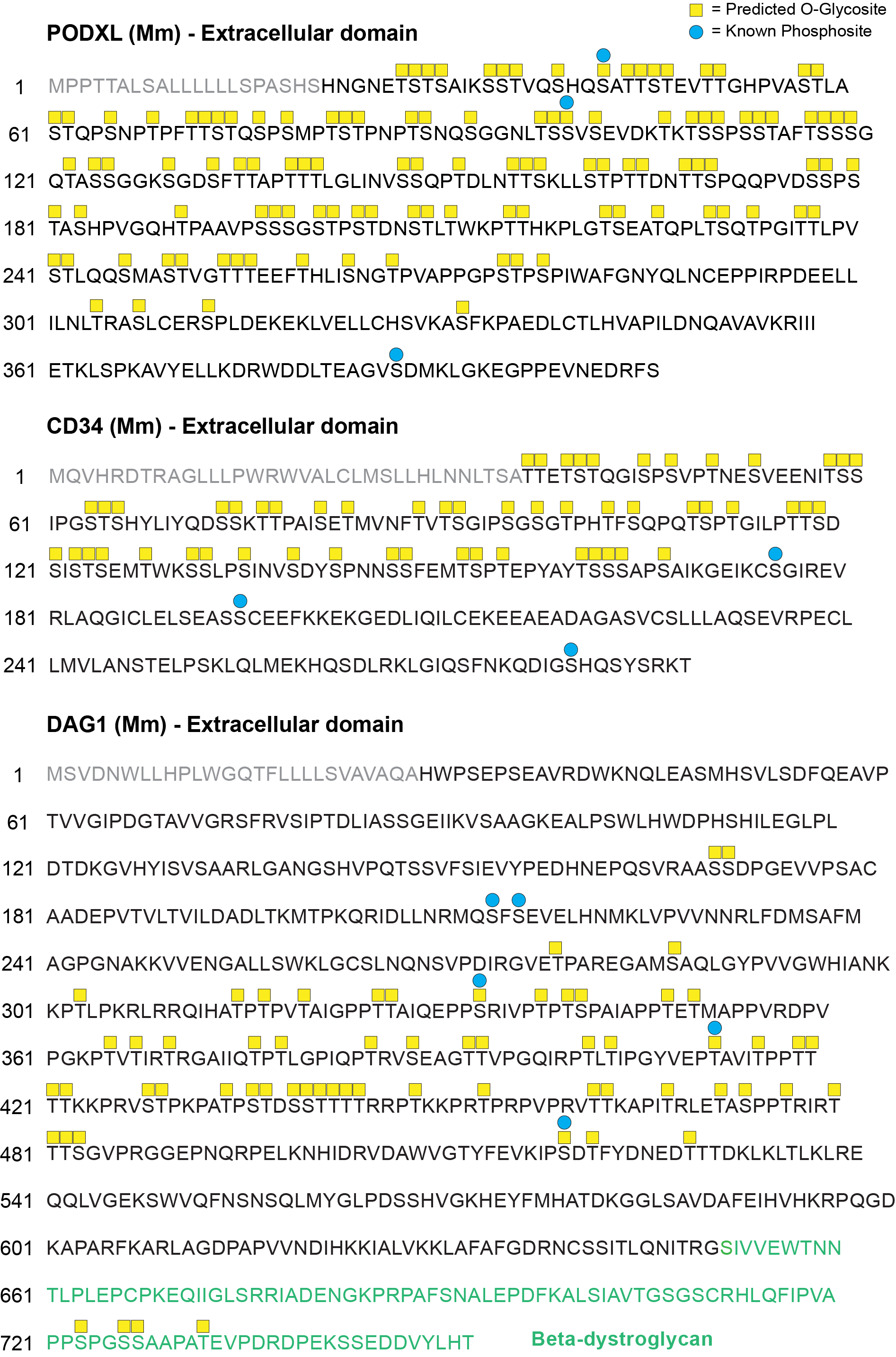
**


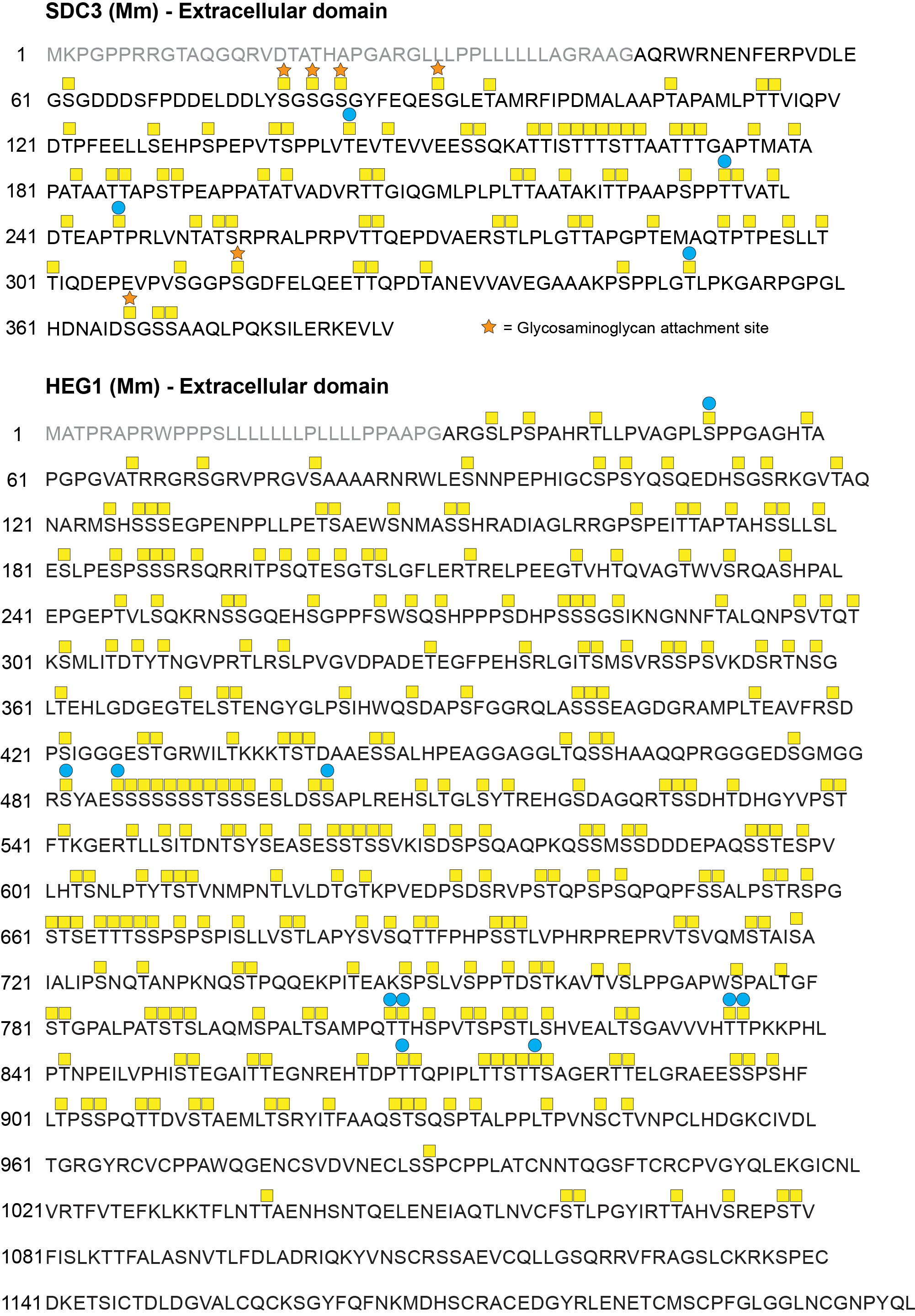


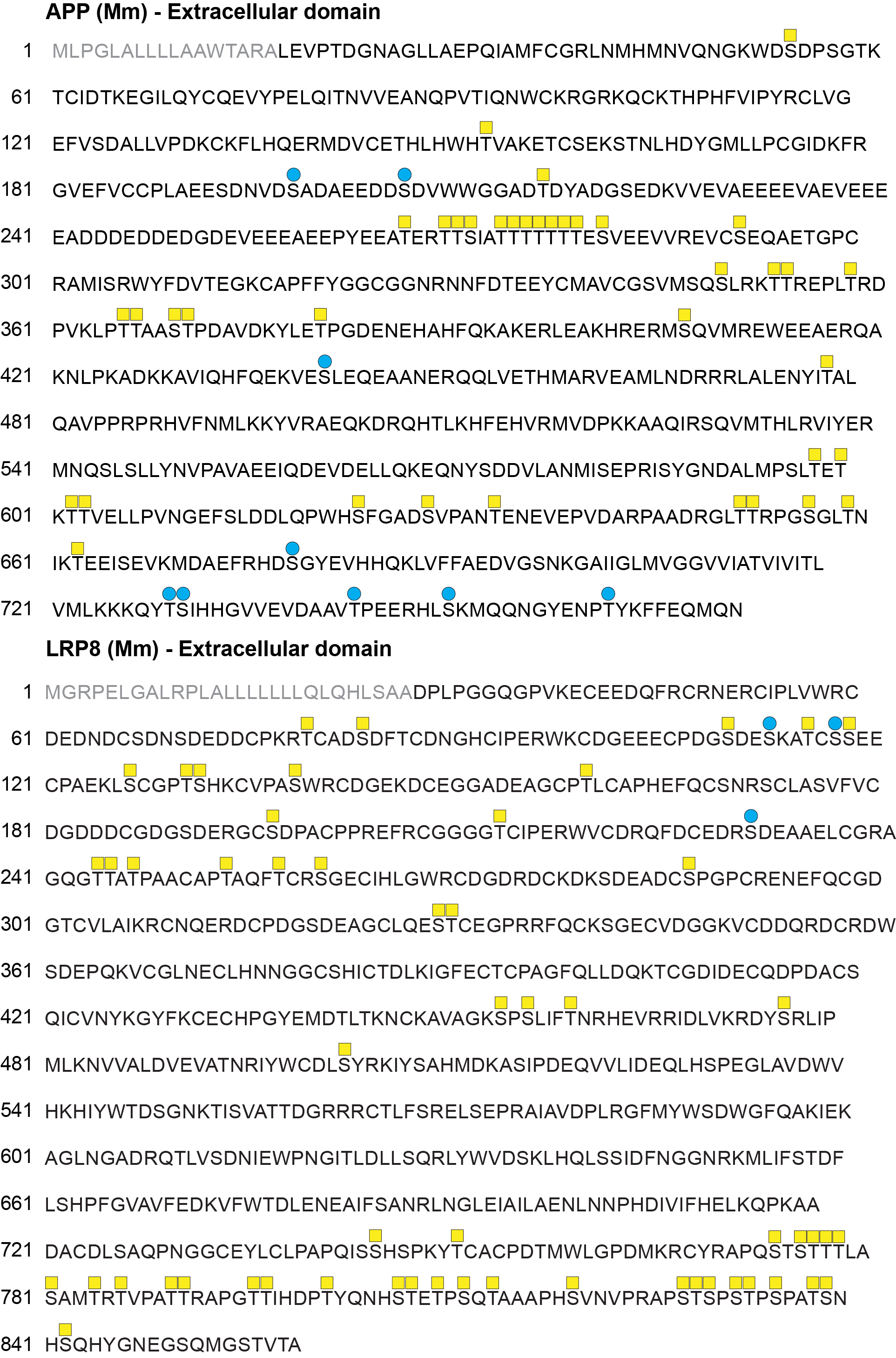


**Supplementary Figure 1. Examples of brain endothelial mucin-domain glycoproteins.**

Annotation of predicted O-glycosites^5^ (yellow squares) and known phosphosites^6^ (blue circles) in the extracellular domains of various mucin-domain glycoproteins expressed in brain endothelial cells. **Supplementary Figure 2.**

**
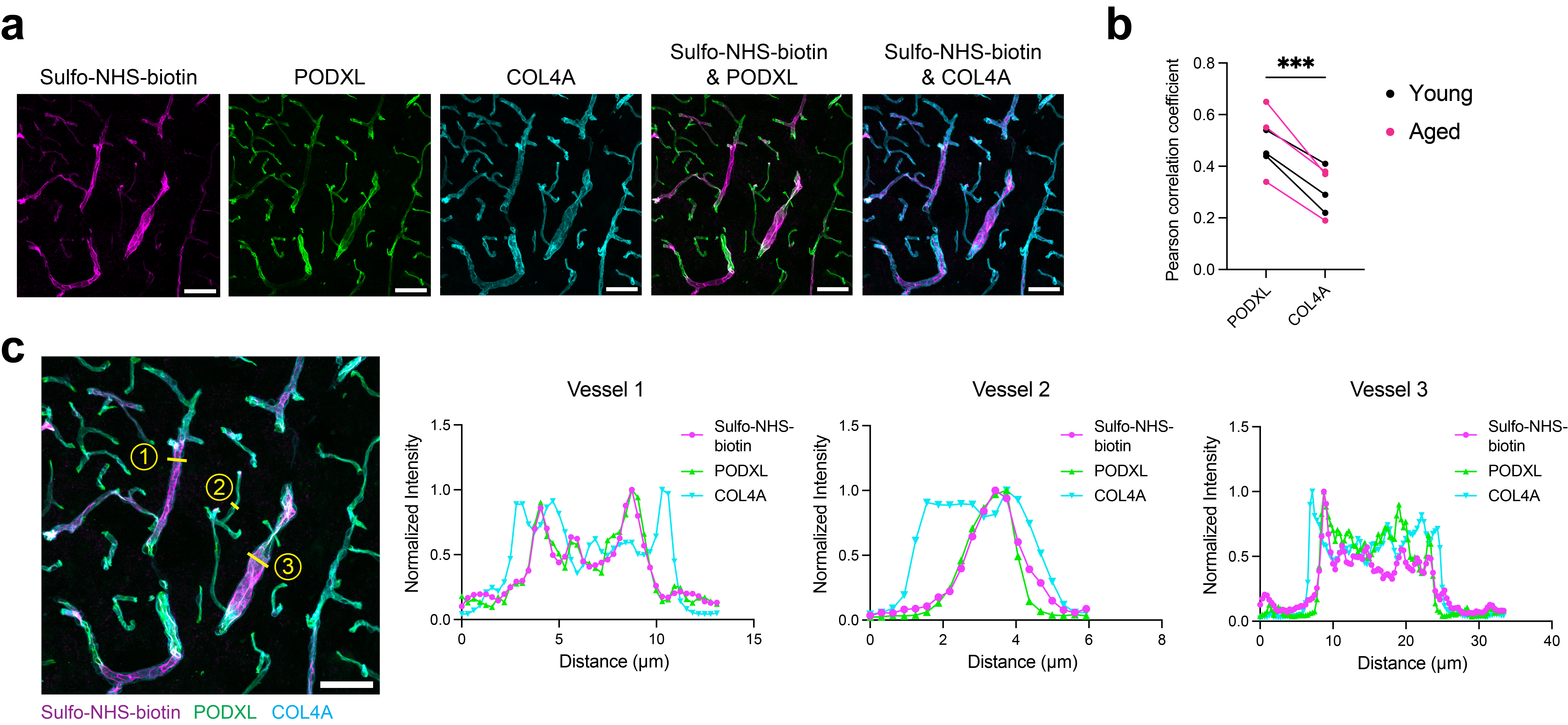
**

**Supplementary Figure 2. Luminally enriched cerebrovascular labeling via sulfo-NHS-biotin perfusion in young and aged mice.**

1. Sulfo-NHS-biotin, PODXL (luminal marker), and COL4A (basement membrane marker) labeling in the cortex of an aged animal used in the luminal cerebrovascular proteomics experiment. Scale bar = 20 µm.
2. Quantification of sulfo-NHS-biotin colocalization with PODXL and COL4A via Pearson correlational analyses (n=3 mice per group; two-sided paired t-test; mean ± s.e.m.).
3. Marker intensity profiles across three vessels showing spatial correlation between sulfo-NHS-biotin, PODXL, and COL4A. Yellow labels indicate where intensity profiles were taken in representative image from aged animal. Scale bar=20 µm.

**Supplementary Figure 3.**

**b**

**a**

**
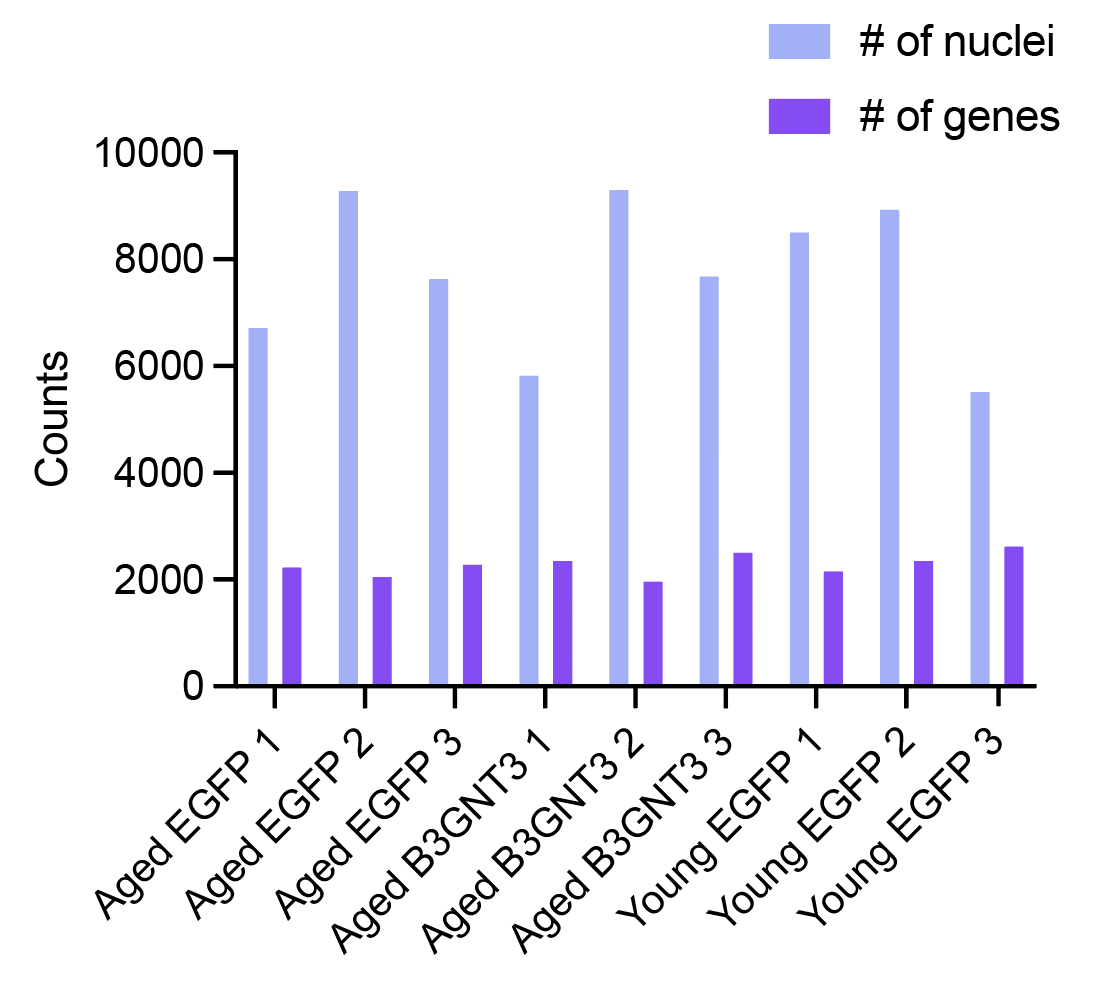
** ****

**c**

**d**

**Supplementary Figure 3. Quality control measures of snRNA-seq samples**

1. Summary of number of nuclei and genes per sample (n=3 animals per group).
2. Violin plots of the number of unique genes identified per nuclei in each experimental group.
3. Violin plots of the number of RNA counts per nuclei in each experimental group.
4. Violin plots of the percentage of mitochondrial transcripts per nuclei in each experimental group.

**Supplementary Figure 4.**

**a**

**
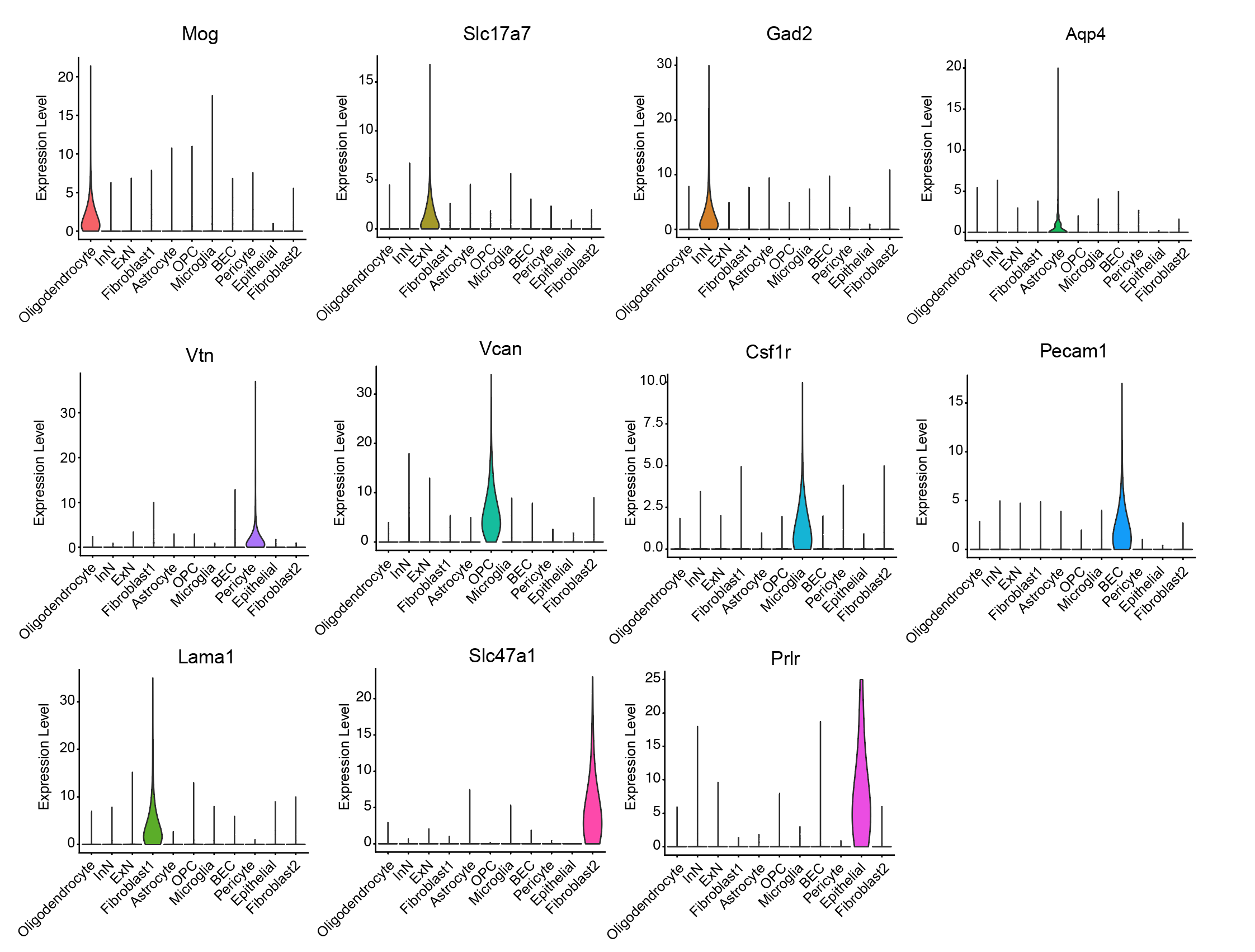
**

**Supplementary Figure 4. Expression of cell markers in each major cell type cluster.**

a) Violin plots of cell marker expression in each major cell type cluster based on log normalized counts.

**References:**

1. Reitsma, S., Slaaf, D. W., Vink, H., Van Zandvoort, M. A. M. J. & Oude Egbrink, M. G. A. The endothelial glycocalyx: Composition, functions, and visualization. *Pflugers Arch. Eur. J. Physiol.* **454**, 345–359 (2007).
2. Reed, M. J. *et al.* Microvasculature of the Mouse Cerebral Cortex Exhibits Increased Accumulation and Synthesis of Hyaluronan With Aging. J. Gerontol. A. Biol. Sci. Med. Sci*.* **72** (2017).
3. Nardis C. D. *et al.* Recombinant Expression of the Full-length Ectodomain of LDL Receptor-related Protein 1 (LRP1) Unravels pH-dependent Conformational Changes and the Stoichiometry of Binding with Receptor-associated Protein (RAP). *JBC* **292** (2017).
4. Anggara, K. *et al.* Direct observation of glycans bonded to proteins and lipids at the single-molecule level. *Science* **382** (2023).
5. Steentoft, C. *et al.* Precision mapping of the human O‐GalNAc glycoproteome through SimpleCell technology. *EMBO J.* **32** (2013).
6. Hornbeck, P. V. *et al.* PhosphoSitePlus, 2014: mutations, PTMs and recalibrations. *Nucleic Acids Res.* **43** (2015).
