# Supplementary material for: Glycocalyx dysregulation impairs blood–brain barrier in ageing and disease
Source: Nature. 2025 Feb 26;639(8056):985–94. doi: 10.1038/s41586-025-08589-9 (PMC11946907; doi:10.1038/s41586-025-08589-9)
Supplement: Supplementary file 2 — Reporting Summary [file 41586_2025_8589_MOESM2_ESM.pdf]

nature portfolio

Corresponding author(s): Tony Wyss-Coray; Carolyn R. Bertozzi

Last updated by author(s): Dec 13, 2024

Reporting Summary

Nature Portfolio wishes to improve the reproducibility of the work that we publish. This form provides structure for consistency and transparency in reporting. For further information on Nature Portfolio policies, see our Editorial Policies and the Editorial Policy Checklist.

Statistics

For all statistical analyses, confirm that the following items are present in the figure legend, table legend, main text, or Methods section.

n/a	Confirmed	
☐	☒	The exact sample size (*n*) for each experimental group/condition, given as a discrete number and unit of measurement
☐	☒	A statement on whether measurements were taken from distinct samples or whether the same sample was measured repeatedly
☐	☒	The statistical test(s) used AND whether they are one- or two-sided<br>*Only common tests should be described solely by name; describe more complex techniques in the Methods section.*
☐	☒	A description of all covariates tested
☐	☒	A description of any assumptions or corrections, such as tests of normality and adjustment for multiple comparisons
☐	☒	A full description of the statistical parameters including central tendency (e.g. means) or other basic estimates (e.g. regression coefficient) AND variation (e.g. standard deviation) or associated estimates of uncertainty (e.g. confidence intervals)
☐	☒	For null hypothesis testing, the test statistic (e.g. *F*, *t*, *r*) with confidence intervals, effect sizes, degrees of freedom and *P* value noted<br>*Give P values as exact values whenever suitable.*
☒	☐	For Bayesian analysis, information on the choice of priors and Markov chain Monte Carlo settings
☐	☒	For hierarchical and complex designs, identification of the appropriate level for tests and full reporting of outcomes
☐	☒	Estimates of effect sizes (e.g. Cohen's *d*, Pearson's *r*), indicating how they were calculated

Our web collection on statistics for biologists contains articles on many of the points above.

Software and code

Policy information about availability of computer code

Data collection	FACS Diva (BD), SH800S (Sony), ZEN Black (Zeiss), Chromium Single Cell 3' Kit v3.1 (10X Genomics), NovaSeq X (Novogene), TimsTOF Pro (Bruker), Q Exactive HF-X (Thermo)
Data analysis	FlowJo (Treestar), Prism 8 and 9 (GraphPad), R (ggplot2, tidyverse, DESeq2, Seurat), ImageJ/ Fiji (NIH), MaxQuant (Max Planck), Perseus (Max Planck), Spectronaut (Biognosys)

For manuscripts utilizing custom algorithms or software that are central to the research but not yet described in published literature, software must be made available to editors and reviewers. We strongly encourage code deposition in a community repository (e.g. GitHub). See the Nature Portfolio guidelines for submitting code & software for further information.

Data

Policy information about availability of data

All manuscripts must include a data availability statement. This statement should provide the following information, where applicable:

- Accession codes, unique identifiers, or web links for publicly available datasets
- A description of any restrictions on data availability
- For clinical datasets or third party data, please ensure that the statement adheres to our policy

Raw mass spectrometry data generated in this study have been deposited in the PRIDE database under accession code PXD043964 and PXD058690. Raw sequencing data have been deposited in the NCBI Gene Expression Omnibus (GEO) under accession codes GSE268263 and GSE268642.

Research involving human participants, their data, or biological material

Policy information about studies with human participants or human data. See also policy information about sex, gender (identity/presentation), and sexual orientation and race, ethnicity and racism.

Reporting on sex and gender	*Use the terms sex (biological attribute) and gender (shaped by social and cultural circumstances) carefully in order to avoid confusing both terms. Indicate if findings apply to only one sex or gender; describe whether sex and gender were considered in study design; whether sex and/or gender was determined based on self-reporting or assigned and methods used.*<br>*Provide in the source data disaggregated sex and gender data, where this information has been collected, and if consent has been obtained for sharing of individual-level data; provide overall numbers in this Reporting Summary. Please state if this information has not been collected.*<br>*Report sex- and gender-based analyses where performed, justify reasons for lack of sex- and gender-based analysis.*
Reporting on race, ethnicity, or other socially relevant groupings	*Please specify the socially constructed or socially relevant categorization variable(s) used in your manuscript and explain why they were used. Please note that such variables should not be used as proxies for other socially constructed/relevant variables (for example, race or ethnicity should not be used as a proxy for socioeconomic status).*<br>*Provide clear definitions of the relevant terms used, how they were provided (by the participants/respondents, the researchers, or third parties), and the method(s) used to classify people into the different categories (e.g. self-report, census or administrative data, social media data, etc.)*<br>*Please provide details about how you controlled for confounding variables in your analyses.*
Population characteristics	Male and female aged (58-84 years old) cognitively normal and clinically-diagnosed Alzheimer's disease patients.
Recruitment	Subjects were not recruited specifically for this study. Samples are derived from a brain bank maintained by the Stanford/ VA ACRC (Aging Clinical Research Center) from patients that provide consent for broad, de-identified data sharing under Institutional Review Board (IRB) approval.
Ethics oversight	Stanford/VA Aging Clinical Research Center (ACRC), Stanford Institutional Review Board (IRB)

Note that full information on the approval of the study protocol must also be provided in the manuscript.

Field-specific reporting

Please select the one below that is the best fit for your research. If you are not sure, read the appropriate sections before making your selection.

☒ Life sciences ☐ Behavioural & social sciences ☐ Ecological, evolutionary & environmental sciences

For a reference copy of the document with all sections, see nature.com/documents/nr-reporting-summary-flat.pdf

Life sciences study design

All studies must disclose on these points even when the disclosure is negative.

Sample size	No power analyses were used to predetermine sample sizes, but sample sizes are similar to those reported in previous publications.
Data exclusions	None.
Replication	For in vivo experiments, biological replicates and independent cohorts of mice were used. Data in Figures 1b-d, 1h-o, 2a-b, 2f-j, 3e-n, 4f-i, and 5j-l and Extended Data Figures 2a, 3f-i, 7a-c, 7h-i, and 8h-k were successfully replicated in at least two independent experiments as stated in the "Statistics and reproducibility" section.
Randomization	For AAV studies, same aged mice were randomly assigned into experimental groups. For other animal studies, cages of young and aged mice at the same veterinary facility were allocated at random for experiments.
Blinding	Immunohistological analyses, nuclei isolations, and behavioral analyses were performed by a blinded observer. In general, experimenters were blinded to group allocation during data acquisition and analysis.

Reporting for specific materials, systems and methods

We require information from authors about some types of materials, experimental systems and methods used in many studies. Here, indicate whether each material, system or method listed is relevant to your study. If you are not sure if a list item applies to your research, read the appropriate section before selecting a response.

Materials & experimental systems

n/a	Involved in the study
☐	☒ Antibodies
☐	☒ Eukaryotic cell lines
☒	☐ Palaeontology and archaeology
☐	☒ Animals and other organisms
☒	☐ Clinical data
☒	☐ Dual use research of concern
☒	☐ Plants

Methods

n/a	Involved in the study
☒	☐ ChIP-seq
☐	☒ Flow cytometry
☒	☐ MRI-based neuroimaging

Antibodies

Antibodies used

Flow cytometry: rat anti-CD31-PE/CF594 (1:100, BD, 563616), rat anti-CD45-PE/Cy7 (1:200, Biolegend, 103114), APC-anti-CD54 (1:100, BioLegend, 116120), FITC-anti-VCAM1 (1:100, Thermo Fisher Scientific, 11-1061-82), AF647-anti-CD62P (1:100, BD Biosciences, 563674), anti-CD62E (1:100, Thermo Fisher Scientific, 14-0627-82), AF647-anti-NeuN (1:100, Abcam, ab190565)

Immunostaining primary antibodies: mouse anti-heparan sulfate (1:100, Amsbio, clone 10E4, 370255-1), mouse anti-chondroitin sulfate (1:100, Sigma, clone CD-56, C8035), biotinylated hyaluronan binding protein (HABP) (1:150, Amsbio, AMS.HKD-BC41), rabbit anti-C1galt1 (1:100, Thermo Fisher Scientific, PA5-52814), rabbit anti-B3gnt3 (1:100, Thermo Fisher Scientific, PA5-21988), goat anti-CD31 (1:100, R&D, AF3628), goat anti-Iba1 (1:100, Abcam, ab5076), rat anti-CD68 (1:100, Bio-Rad, MCA1957), goat anti-collagen type IV (1:100, Sigma, AB769), rat anti-NID1 (1:100, Thermo Fisher Scientific, MA1-06501), rabbit anti-CLDN5 (1:100, Thermo Fisher Scientific, 34-1600), mouse anti-ZO1 (1:100, Thermo Fisher Scientific, 33-9100), goat anti-albumin (1:100, Thermo Fisher Scientific, A90-134A), goat anti-PODXL (1:100, R&D Systems, AF1556), rabbit anti-CAV1 (1:100, Cell Signaling Technologies; 3267S), mouse anti-CLTC (1:100, Thermo Fisher Scientific, MA1-065).

Immunostaining secondary antibodies: Alexa Fluor 488 donkey anti-goat-IgG (1:250; Invitrogen, A-11055); Alexa Fluor 555 donkey anti-rat-IgG (1:250; Invitrogen, A-48270); Alexa Fluor 555 donkey anti-mouse-IgG (1:250; Invitrogen, A-31570); Alexa Fluor 555 donkey anti-goat-IgG (1:250; Invitrogen, A-21432); Alexa Fluor 647 donkey anti-mouse-IgG (1:250; Invitrogen, A-31571); Alexa Fluor 647 donkey anti-rabbit-IgG (1:250; Invitrogen, A-31573)

Validation

All antibodies were validated for the indicated species and applications by the manufacturer.

Eukaryotic cell lines

Policy information about cell lines and Sex and Gender in Research

Cell line source(s)

b.End3 cells (ATCC), HEK293T (Takara)

Authentication

The cell lines were authenticated by the vendors. Immunofluorescence staining of Bend.3 cells for BBB-specific markers of adherens junctions and tight junctions, specifically ß-catenin, Claudin-5, and VECadherin was used to validate the cell line previously in the lab.

Mycoplasma contamination

The cell lines tested negative for mycoplasma contamination.

Commonly misidentified lines
(See ICLAC register)

No commonly misidentified lines were used.

Animals and other research organisms

Policy information about studies involving animals; ARRIVE guidelines recommended for reporting animal research, and Sex and Gender in Research

Laboratory animals

C57Bl/6 male mice, aged (16-21 months from NIA rodent colony), young (3 months from Jackson Labs). All mice were kept on a 12-h light/dark cycle and provided ad libitum access to food and water.

Wild animals

This study did not involve wild animals.

Reporting on sex

Only male mice were used in this study.

Field-collected samples

This study did not involve field-collected samples.

Ethics oversight

Institutional Animal Care and Use Committee at Stanford University; V.A. Palo Alto Committee on Animal Research

Note that full information on the approval of the study protocol must also be provided in the manuscript.

Flow Cytometry

Plots

Confirm that:

☒ The axis labels state the marker and fluorochrome used (e.g. CD4-FITC).

☒ The axis scales are clearly visible. Include numbers along axes only for bottom left plot of group (a 'group' is an analysis of identical markers).

☒ All plots are contour plots with outliers or pseudocolor plots.

☒ A numerical value for number of cells or percentage (with statistics) is provided.

Methodology

Sample preparation	Mouse brain microvessels were dissociated enzymatically (papain, collagenase/dispase) or mechanically (dounce, strainer) into single cells which were stained on ice in PBS supplemented with 1% bovine serum albumin and passed through a 100-micron strainer before flow cytometry.<br>bEnd.3 cells were detached from plates using enzyme-free cell dissociation buffer and stained for 30 minutes on ice in PBS supplemented with 1% bovine serum albumin and passed through a 100-micron strainer before flow cytometry.
Instrument	BD LSRFortessa; Sony SH800S
Software	BD FACS Diva, Cytobank, FlowJo
Cell population abundance	For glyco-profiling, at least 7,000 brain endothelial cells were analyzed.<br>For bEnd.3 experiments, at least 20,000 GFP+ cells were analyzed.
Gating strategy	Positive and negative gates were set using unstained and fluorescence minus one (FMO) background intensity controls.

☒ Tick this box to confirm that a figure exemplifying the gating strategy is provided in the Supplementary Information.
